# A Computational Investigation of the ^15^N Chemical Shift Behavior of *Strychnos* Alkaloids

**DOI:** 10.3390/ijms27093840

**Published:** 2026-04-26

**Authors:** Valentin A. Semenov, Leonid B. Krivdin, Gary E. Martin

**Affiliations:** 1 A. E. Favorsky Irkutsk Institute of Chemistry, Siberian Branch of the Russian Academy of Sciences, Favorsky St. 1, 664033 Irkutsk, Russia; semenovval@inbox.ru (V.A.S.); krivdin55@gmail.com (L.B.K.); 2 Department of Chemistry and Biochemistry, Seton Hall University, 400 South Orange Ave., South Orange, NJ 07079, USA

**Keywords:** alkaloids of *Strychnos*-type, stereochemistry, conformational analysis, ^15^N NMR, DFT calculations, vinorelbine

## Abstract

Members of the broad family of *Strychnos* alkaloids have been the favorite molecules for the development and evaluation of new NMR methods for many years, including the establishment of the ^1^H-^15^N two-dimensional NMR methods. The present study is an effort to computationally evaluate the ^15^N chemical shift behavior of eight members of this structurally diverse group of indole alkaloids. The molecules range from relatively “simple” strychnine and brucine and their respective *N*-oxides to molecules as complex as vinorelbine, a synthetic analog used in cancer chemotherapy, and sungucine, a naturally occurring complex dimeric member of the family. The 20 ^15^N chemical shifts in this study afforded a CMAE of 2.96 ppm and an RMSD of 3.50 ppm, with the data affording a correlation coefficient, *r* = 0.997.

## 1. Introduction

“Strychnine!” Woodward began a seminal account of the total synthesis of the complex indole alkaloid strychnine published in *Tetrahedron* [1] in 1963 with that single exclamation. In an earlier paper Woodward referred to the strychnine using the following quote: “The tangled skein of atoms which constitutes its molecule provided a fascinating structural problem which was pursued intensively during the century just past, and was solved finally only within the last decade” [2]. All of this work was, of course, before the development of modern pulsed NMR techniques and far preceded the development of two-dimensional (2D) NMR techniques that are now routinely employed to solve complex chemical structure problems. Well, after the development of 2D NMR methods that routinely focus on the ^1^H and ^13^C nuclei, it became possible to study ^15^N at natural abundance [3,4]. In the intervening years, numerous reviews and chapters appeared on various aspects and applications of ^15^N NMR in small molecules including alkaloids by a number of authors, to which readers are referred for more details [5,6,7,8,9,10,11,12,13].

Strychnine has been employed for many years by one of the authors of this paper as a test compound for the development and evaluation of new heteronuclear ^1^H-^13^C, ^1^H-^15^N, and homonuclear ^13^C-^13^C NMR two-dimensional (2D) techniques. The compound is readily available globally, which allowed authors to directly reproduce reported experimental results using new NMR experiments in their own laboratories prior to embarking on the elucidation of unknown structures using such methods. Thus, the choice of strychnine (**1**) as a model compound for early investigations of long-range ^1^H-^15^N HMBC was a logical choice. More recently, with the development of the LR-HSQMBC experiment in 2014 [14], even longer-range ^1^H-^15^N correlations that would be obliterated by the magnitude calculation processing used with HMBC data can be observed [15].

**Scheme 1:**
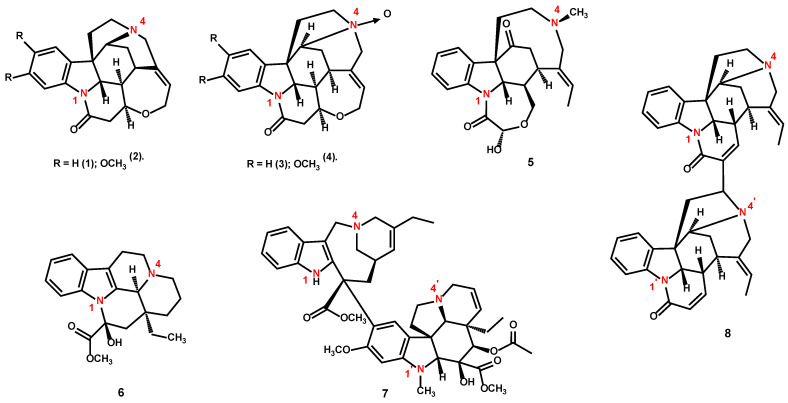
Structures and enumeration of the studied *Strychnos* alkaloids: strychnine (**1**); brucine (**2**) and their respective *N*-oxides (**3** and **4**); holstiine (**5**); vincamine (**6**); vinorelbine (**7**); and sungucine (**8**).

Given the importance of nitrogen in molecules ranging from pharmaceuticals to alkaloids, it was appropriate to initiate an investigation into the applicability of ^15^N chemical shift calculation methodology for the prediction of ^15^N chemical shifts. Taking into account the frequent usage of *Strychnos* alkaloids as model compounds for the development and evaluation of new two-dimensional (2D) NMR experiments, these were again a logical choice for this investigation. The ensemble of *Strychnos* alkaloids used in this investigation is shown in Scheme 1.

## 2. Results and Discussion

The interpretation of the NMR spectra of *Strychnos* alkaloids, each containing several asymmetric centers, is a difficult and sometimes challenging task. In the series of *Strychnos* alkaloids, **1**–**8**, investigated in this study, there are a large number of hydrogen and carbon atoms that have a similar stereoelectronic environment and, as a result, very similar or even equivalent NMR chemical shifts. Very often, ^1^H NMR spectra of such alkaloids present superposition of several individual multiplets of the second or even higher order providing complicated unresolved patterns, which are sometimes very difficult to analyze. This is even more difficult in the light of magnetic nonequivalence of protons located in the *axial* and *equatorial* positions of condensed cyclic compounds, the latter providing the general constitution of monoterpene indole alkaloids. Unambiguous interpretation of NMR spectra is a guiding thread in the spectral interpretation of natural products using an array of homo- and heteronuclear 2D-NMR techniques [13,16,17], including techniques like the newly reported i-HMBC experiment [18] and anisotropic NMR methods to resolve stereochemical concerns [19,20,21,22].

In some cases, certain spectral inconsistencies and ambiguities may occur that can be resolved only using heteronuclear correlations involving the ^15^N nucleus. The knowledge of characteristic ranges of ^15^N NMR chemical shifts obtained using high-level theoretical calculations could significantly simplify this task, as demonstrated in this paper. The present study continues our series of papers [23,24,25,26] dealing with the stereochemical analysis of natural bisindole alkaloids.

**Figure 1:**
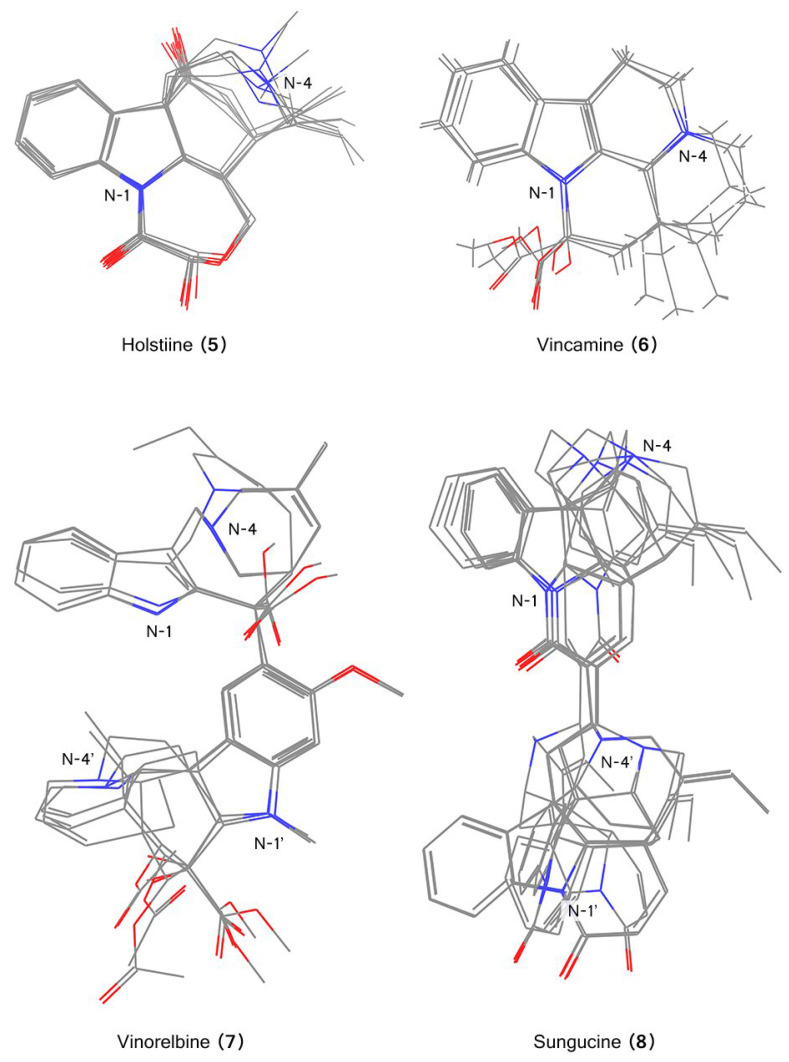
Conformational behavior and main conformer groups of the studied *Strychnos* alkaloids **5**–**8**.

At the initial stage of this study, we performed a conformational scrutiny of **1**–**8**, which revealed several groups of low-energy conformers for each of the alkaloids from this series. Details of the conformational search, optimization of geometric parameters, and calculation of shielding constants are provided in Materials and Methods. The spatial structures of the most interesting low-energy groups of conformers of compounds **5**–**8** are presented in Figure 1, while Cartesian coordinates of all calculated structures are given in Supplementary Materials.

It is well known that averaged ^1^H NMR signals in solution are determined by a set of the most probable conformers [27,28]. Therefore, as a rule, when modeling NMR chemical shifts, the values of shielding constants weighted according to the Boltzmann distribution are used with individual coefficients depending on the free energy of each of the conformers. However, it has recently been convincingly shown that even the prototypically rigid strychnine **1** exhibits conformational dynamics on the NMR timescale [29]. Therefore, for the monomeric alkaloids **1**–**6**, the averaged structural parameters resulting from this rapid exchange are well known, and we relied on the known averaged conformations, which provide an appropriate basis for ^15^N chemical shift calculations. Since the proportion of minor conformers is typically less than 10%, their contribution to the averaged ^15^N chemical shift is within the computational error. For this reason, in this study, no Boltzmann averaging of ^15^N NMR chemical shifts was performed, and the saturated ring conformations in compounds **1**–**6** were predetermined when constructing the spatial structures of the molecules. The conformational distribution of compounds **7** and **8** primarily represents the distribution of their static rotamers around the C-16′–C-17′ bond, as shown in Figure 1.

Calculations of the free energies of those rotamers performed at the M06-2X/pecG-2 level have shown that corresponding rotation barriers were up to 15 kcal/mol. According to the Eyring Equation (1), the lifetime of each rotamer at 298 K is about 16 ms. The difference in the ^15^N chemical shifts in various rotamers is up to 30 ppm, which at a frequency of 41 MHz (at *B*_0_ = 9.4 T) for ^15^N corresponds to Δ*ν* ≈ 1200 Hz. At the same time, the actual lifetimes of rotamers are an order of magnitude higher than the threshold of rapid exchange in the ^15^N NMR time scale (being 0.13 ms), which means that no conformational averaging exists. Each rotamer gives individual, non-averaging signals, and the experimental spectrum is a superposition of the spectra of all rotamers present in the sample [27]. Thus, a standard Boltzmann averaging procedure, which assumes rapid interconversion and is routinely applied to flexible systems, is physically inapplicable to the studied alkaloids **7** and **8**. It follows that there is no point in performing correlation analysis of calculated NMR chemical shifts for *untrue* conformers.

It should also be noted that for molecules of this size, i.e., having more than 40–50 non-hydrogen atoms, the systematic error in calculating the relative free energies at the DFT level, which is of the order of 0.5–1.0 kcal/mol, is comparable to the difference in energy that could lead to a significant redistribution of populations, or even exceed them. In this regard, instead of the mathematically unjustified Boltzmann averaging, we applied here a strategy of the “manual” selection of rotamers, with the main criterion of the best correlation between calculated and experimental ^15^N NMR chemical shifts, rather than thermodynamic reasoning. Calculated ^15^N NMR chemical shifts in all found low-energy conformers of **1**–**8** are provided in Table S1, see Supplemental Materials.

In the next step, geometric parameters of all groups of favorable conformers in the series of all alkaloids **1**–**8** were refined using the higher-level optimization within the framework of DFT using the Minnesota functional M06-2X. To describe atomic orbitals, we used our newest basis set pecG-2. This basis set was specially optimized to find the most accurate equilibrium geometry by taking into account interatomic bond lengths.

Next, for the optimized structures of all conformers in the series of **1**–**8**, the shielding constants together with the corresponding NMR chemical shifts were calculated within the GIAO-PBE0/pecS-2//aug-pecS-2 scheme. Calculation of shielding constants was performed using effective basis sets of the pecS-2 family, optimized for calculations of large synthetic organic molecules and natural products. Since for the nitrogen atom it is necessary to more accurately account for the atomic orbitals describing its lone pair, in this case, the aug-pecS-2 basis set was used, the latter extended by diffuse functions to describe the regions of spin density being far from the nucleus, see Materials and Methods (Part 3). The influence of solvation effects, which are particularly pronounced for ^15^N NMR chemical shifts, was accounted for using the framework of Tomasi’s Polarizable Continuum Model, IEF-PCM.

**Figure 2:**
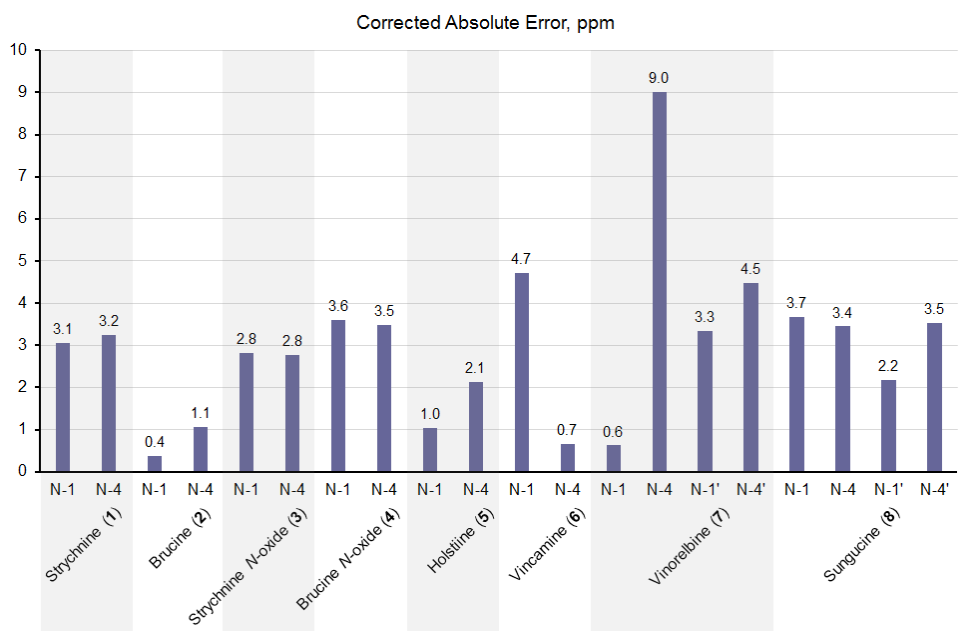
Distribution diagram of absolute deviations of ^15^N NMR chemical shifts, calculated at the (GIAO-DFT)PBE0/aug-pecS-2 level for alkaloids **1**–**8**.

Calculated ^15^N NMR chemical shifts were then used for the correlation with known experimental data. For establishing the stereochemistry of **1**–**8**, we applied our general workflow, originally proposed for the analysis of stereochemically rich natural products [30]. This estimation was performed for all conformers of compounds **1**–**8** using the well-known statistical descriptors, such as Corrected Mean Absolute Error (CMAE), Root Mean Square Deviation (RMSD), and Pearson correlation coefficient, evaluated accordingly with the Equations (2)–(4), see Materials and Methods. The results of the performed calculations based on CMAE are exemplified in Figure 2.

As shown by these data, a good correlation was obtained between the calculated and the experimental ^15^N NMR chemical shifts for **1**–**8**. The average absolute deviation was found to be in the range of 0.7–4.5 ppm, which is a very good result compared with the data we obtained previously for small nitrogen-containing organic compounds [31,32,33]. The only questionable issue concerns the interpretation of the signal, which can be assigned to the N-4 atom in vinorelbine (**7**); this point will be discussed in more detail below.

Table 1 shows the calculated ^15^N NMR chemical shifts for each nitrogen atom in compounds **1**–**8**, as compared to available experimental data. For clarity, relative deviations are given with their signs taken into account.

It should also be noted that for all eight *Strychnos*-type analogs studied in this paper, the established absolute configurations of their asymmetric centers located on carbons and nitrogens were found to be in good agreement with the present results. As has already been mentioned, the only clearly identified ambiguity was associated with the signal of the N-4 atom in vinorelbine (**7**). Since the N-4 atom in the quinolizidine C/D system of *Strychnos* alkaloids almost always adopts the 4*R* configuration, in the present study, we considered that the eight-membered C/E ring “inherited” the same 4*R* configuration from the quinolizidine C/D system. However, for all of the calculated low-energy conformers **7a**–**c** (see Supporting Information for coordinates), the upfield deviation of the N-4 chemical shift is about +(9.0–9.9) ppm. An important clarification to be made here is that the absence of conformational averaging between rotamers around the C-16′–C-17′ bond does not at all imply the absence of low-frequency dynamic vibrations *in the framework of stable conformations* of the eight-membered C/E ring within each of the rotamers of **7**. The C/E ring exhibits fast, low-barrier bending motions occurring on the 10^−9^–10^−12^ second range of the NMR time scale. A stable-state DFT calculation searching for a single minimum on the PES does not adequately estimate this type of fast averaging. We believe that this limitation likely explains the persistent upfield bias of the calculated N-4 chemical shift in all of the C/E conformations **a**–**c** of **7**.

To exclude the possibility of an incorrect assignment of the N-4 atom configuration in vinorelbine **7**, we attempted to optimize the 4*S* diastereomer at the M06-2X/pecG-2 level. All optimization attempts converged on the 4*R* configuration, indicating that the 4*S* diastereomer does not correspond to a local minimum on the PES. This is consistent with the severe steric constraints imposed by the eight-membered C/E ring and with the biogenetic hypothesis, according to which the N-4 atom of the C/D quinolizidine system of *Strychnos* alkaloids usually adopts the 4*R* configuration. Therefore, the consistent upfield bias of the calculated N-4 chemical shift is indeed explained by the dynamic lability of the eight-membered ring and not by an error in the determination of the N-4 configuration.

**Figure 3:**
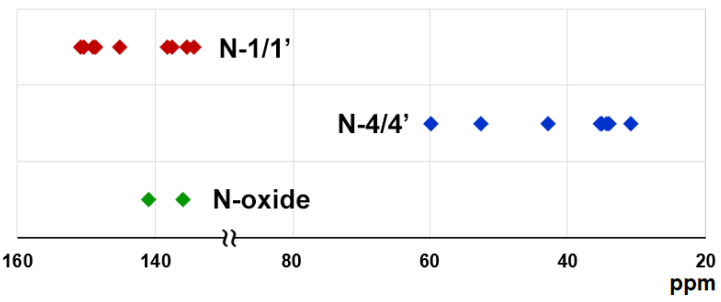
Characteristic ranges of calculated ^15^N NMR chemical shifts for particular nitrogen types in *Strychnos* alkaloids **1**–**8**.

Further, based on the calculated ^15^N NMR chemical shifts in alkaloids **1**–**8**, a diagram showing the characteristic ranges of nitrogen shifts was created for the nitrogen atoms N-1/1′, N-4/4′, as well as for the oxide nitrogen atom in the N⟶O stereoelectronic state (see Figure 3). Overall, despite the fairly “predictable” nature of the high-field nitrogen atom of the amine-type N-4/4′, the scatter of ^15^N NMR chemical shift values was more than 30 ppm, which warrants a more detailed study.

**Figure 4:**
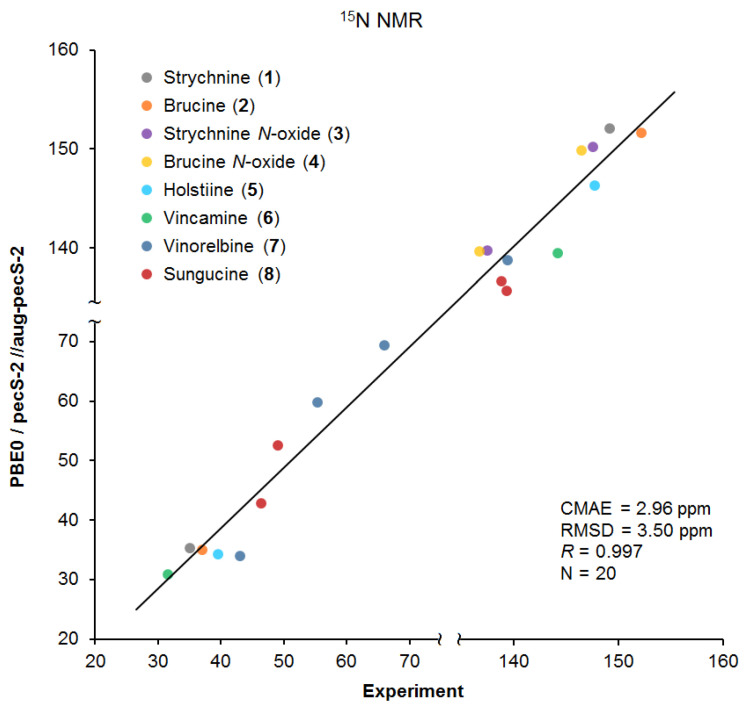
Correlation plot of calculated vs. experimental ^15^N NMR chemical shifts (ppm) of **1**–**8**. The points corresponding to each compound are represented by different colors provided in the plots. All correlation parameters are evaluated for the entire series of **1**–**8**.

As a final illustration, Figure 4 displays a correlation graph of experimental and calculated ^15^N NMR chemical shifts in the series of **1**–**8**. These results demonstrate acceptable accuracy of the proposed calculation protocol for the stereochemical analysis of natural *Strychnos*-type alkaloids. The observed integral errors CMAE (evaluated here for the most probable conformers) do not exceed 3 ppm, which is approximately 2% of the entire range of the observed ^15^N NMR chemical shifts. The RMSD is 3.5 ppm, and the correlation coefficient is very close to one. The complete set of experimental and calculated ^15^N NMR chemical shifts is provided in Table S1, see Supporting Information.

## 3. Materials and Methods

### 3.1. Conformational Search and Optimization of Geometric Parameters

The initial conformational search of alkaloids **1**–**8** was performed using the OPLS3 force field in the liquid phase of a particular solvent, employing the MacroModel module implemented in the Schrödinger Maestro package [38]. During this search, approximately 10^5^ steps were performed to identify the most probable conformers/rotamers. Subsequently, the unique conformations of the alkaloids were identified and subjected to further geometry optimization using the Gaussian 09 code [39] at the M06-2X/pecG-2 level [40,41]. In this study, we utilized new, efficient pecG-2 basis sets of atomic orbital exponentials for geometric parameter optimization. These basis sets were generated in our laboratory using the simultaneous property-energy-consistent (PEC) algorithm [42], which aims to minimize the molecular energy gradient relative to interatomic bond lengths. The performance of these basis sets was previously tested using both theoretical data and experimental gas-electron diffraction data relative to other popular basis sets commonly used for geometry optimization of molecular structures, including natural products [43,44]. The solvation effect of each particular solvent was accounted for using the IEF-PCM model developed by Tomasi [45,46].

### 3.2. Modeling (Calculation) of Shielding Constants

Calculations of ^15^N NMR isotropic magnetic shielding constants and corresponding chemical shifts were carried out at the GIAO-DFT level in the liquid phase of a particular solvent using the Gaussian 09 program. In these calculations, we employed the one-parameter hybrid functional PBE0 [47], which was used in combination with Rusakov’s basis sets aug-pecS-2 on the nitrogen atoms and pecS-2 on the rest of the atoms [48,49,50]. The solvation effect of each particular solvent in all NMR calculations was taken into account within the IEF-PCM model.

To take into account systematic errors of calculated chemical shifts, we have established correlations between their experimental chemical shifts (*x*) and isotropic magnetic shielding constants (*y*), which were further used to find the linear correlation equations of the *y* = *a*x + *b* type. The parameters *a* and *b* were then used for recalculating theoretical chemical shifts using the equation *δ_recalc_* = (*σ_calc_* − *b*)/*a*.

The average lifetimes of conformers were calculated as the reciprocal of the rate constant of the interconformational transition:

(1) $$\tau =\;\frac{1}{k}=\frac{1}{\frac{k_{B}T}{h}\cdot e^{-\frac{∆G^{\neq }}{RT}}}$$

where *k* is the rate constant of the interconformational transition; *k*_B_ is the Boltzmann constant; *T* is the temperature at observed transition conditions; *h* is Planck’s constant; Δ*G*^≠^ is the free energy of activation of an interconformational transition; *R* is the universal gas constant.

Corrected Mean Absolute Errors (CMAE) were calculated as:

(2) $$CMAE=\;\frac{\sum_{i=1}^{n}\left|\delta_{exp}-\left(\frac{\sigma_{calc}-b}{a}\right)\right|}{n}$$

where *σ_calc_* are the unscaled shielding constants for each of the *n* nuclei in the molecule, while *a* and *b* are the slope and intercept of the linear regression *σ_calc_* = *aδ_exp_* + *b*.

The Root-Mean-Square Deviations (RMSD) were evaluated as:

(3) $$RMSD=\sqrt{\frac{\sum_{i=1}^{n}{\left(\delta_{exp}-\delta_{calc}\right)}^{2}}{n}}$$

where *δ_exp_* and *δ_calc_* are accordingly, experimental and scaled chemical shifts in each of the *n* nuclei.

The Pearson correlation coefficients *r* were calculated as

(4) $$r{(\delta }_{exp},\delta_{calc})=\frac{\sum_{i=1}^{n}\left(\left(\delta_{exp}-\frac{\sum_{i=1}^{n}\delta_{exp}}{n})(\delta_{calc}-\frac{\sum_{i=1}^{n}\delta_{calc}}{n}\right)\right)}{\sqrt{\sum_{i=1}^{n}{\left(\delta_{exp}-\frac{\sum_{i=1}^{n}\delta_{exp}}{n}\right)}^{2}\sum_{i=1}^{n}{\left(\delta_{calc}-\frac{\sum_{i=1}^{n}\delta_{calc}}{n}\right)}^{2}}}$$

where *δ_exp_* and *δ_calc_* are the experimental and scaled NMR chemical shifts in each of the *n* nuclei, respectively.

## 4. Conclusions

In the present study, we have computationally evaluated the behavior of ^15^N NMR chemical shifts in eight members of the structurally diverse group of indole alkaloids, ranging from relatively simple strychnine and brucine and their *N*-oxides to much larger molecules vinorelbine and sungucine. For this purpose, a strategy of the “manual” selection of rotamers, with the main criterion of the best correlation between calculated and experimental ^15^N NMR chemical shifts, was applied rather than thermodynamic reasoning.

A very good correlation was obtained between the calculated and experimental ^15^N NMR chemical shifts in the series of **1**–**8**. The average absolute deviation was found to be in the range of 0.7–4.5 ppm, which is a very good result compared with the data we obtained previously for small nitrogen-containing organic compounds. It followed that observed integral errors CMAE (evaluated for the most probable conformers of this series) did not exceed 3 ppm, which is approximately 2% of the entire range of observed ^15^N NMR chemical shifts. At that, the RMSD was 3.5 ppm.

The only clear ambiguity was associated with the signal of the N-4 atom in vinorelbine (**7**). For all of the calculated low-energy conformers of this alkaloid, the upfield deviation of the N-4 chemical shift was found to be of about +(9.0–9.9) ppm. We believe that this is due to the high dynamical mobility of the eight-membered C/E ring, which cannot be fully taken into account when calculating the stable states. Despite the fairly predictable nature of the high-field nitrogen atom of the amine-type N-4/4′, the range of ^15^N NMR chemical shifts was found to exceed 30 ppm.

## Figures and Tables

**Table 1:** ^15^N NMR chemical shifts (ppm) of **1**–**8**, calculated at the GIAO-PBE0/aug-pecS-2 level as compared to experiment.

Compound	Nitrogen ^a^	Chemical Shifts		Deviation
		Calculated	Experimental ^b^	
Strychnine (**1**)	N-1	150.8	148.0	−2.8
	N-4	35.2	35.0	−0.2
Brucine (**2**)	N-1	150.4	151.0	+0.6
	N-4	35.1	37.0	+1.9
Strychnine *N*-oxide (**3**)	N-1	149.0	146.4	−2.6
	N-4	138.6	136.3	−2.3
Brucine *N*-oxide (**4**)	N-1	148.6	145.3	−3.3
	N-4	138.5	135.5	−3.0
Holstiine (**5**)	N-1	145.1	146.5	+1.4
	N-4	34.3	39.5	+5.2
Vincamine (**6**)	N-1	138.3	143.0	+4.7
	N-4	30.8	31.5	+0.7
Vinorelbine (**7**)	N-1	137.6	138.2	+0.6
	N-4	34.0	43.0	+9.0
	N-1′	69.3	66.0	−3.3
	N-4′	59.8	55.3	−4.5
Sungucine (**8**) ^c^	N-1	134.4	138.1	+3.7
	N-4	52.5	49.1	−3.4
	N-1′	135.4	137.6	+2.2
	N-4′	42.9	46.4	+3.5

^a^ See Scheme 1 for the enumeration of nitrogen atoms. ^b^ Experimental data are taken from references [34,35,36,37]. ^c^ Sungucine (8) ^15^N chemical shift data are reported here for the first time.

## Data Availability

The original contributions presented in this study are included in the article and Supplementary Materials. Further inquiries can be directed to the corresponding author.

## Supplementary Materials

The following supporting information can be downloaded at: https://www.mdpi.com/article/10.3390/ijms27093840/s1.
