# Supplementary material for: A Computational Investigation of the 15N Chemical Shift Behavior of Strychnos Alkaloids
Source: Int J Mol Sci. 2026 Apr 26;27(9):3840. doi: 10.3390/ijms27093840 (PMC13164246; doi:10.3390/ijms27093840)
Supplement: Supplementary file 1 [file ijms-27-03840-s001.zip › ijms-4269717-supplementary.pdf]

SUPPORTING INFORMATION

A Computational Investigation of the ^{15}N Chemical Shift Behavior of *Strychnos* Alkaloids

by

Valentin A. Semenov,[1] Leonid B. Krivdin[1] and Gary E. Martin[2,*]

[1] *A. E. Favorsky Irkutsk Institute of Chemistry, Siberian Branch of the Russian Academy of Sciences, Favorsky St. 1, 664033 Irkutsk, Russia*

[2] *Department of Chemistry and Biochemistry, Seton Hall University, 400 South Orange Ave., South Orange, NJ 07079, USA.*

* *Correspondence: gary.martin1@shu.edu*

TABLE OF CONTENTS

Table S1. ^{15}N NMR chemical shifts (ppm) of main conformers of *Strychnos* alkaloids **1-8**, calculated at the PBE0/aug-pecS-2 level in the liquid phase of particular solvent, modulated within the IEF-PCM scheme. S3

Cartesian coordinates (Angstroms) of main conformers of *Strychnos* alkaloids **1-8**, optimized at the M06-2X/pecG-2 level in the liquid phase of particular solvent, modulated within the IEF-PCM scheme: S4

Compound: **1a** S4

Compound: **2a** S6

Compound: **3a** S8

Compound: **4a** S10

Compound: **5a** S12

Compound: **5b** S14

Compound: **5c** S16

Compound: **5d** S18

Compound: **5e** S20

Compound: **5f** S22

Compound: **6a** S24

Compound: **6b** S26

Compound: **6c** S28

Compound: **7a** S30

Compound: **7b** S33

Compound: **7c** S36

Compound: **8a** S39

Compound: **8b** S42

Compound: **8c** S45

Compound: **8d** S48

Compound: **8e** S51

Compound: **8f** S54

Table S1. ^{15}N NMR chemical shifts (ppm) of main conformers of *Strychnos* alkaloids **1-8**, calculated at the PBE0/aug-pecS-2 level in the liquid phase of particular solvent, modulated within the IEF-PCM scheme.

Compound	Nuclei	Calculated	Experimental
Strychnine (**1**)	N-1	150.8	148.0
	N-4	35.2	35.0
Brucine (**2**)	N-1	150.4	151.0
	N-4	35.1	37.0
Strychnine *N*-oxide (**3**)	N-1	149.0	146.4
	N-4	141.0	136.3
Brucine *N*-oxide (**4**)	N-1	148.6	145.3
	N-4	136.0	135.5
Holstiine (**5**)	N-1	145.1	146.5
	N-4	34.3	39.5
Vincamine (**6**)	N-1	138.3	143.0
	N-4	30.8	31.5
Vinorelbine (**7**)	N-1	137.6	138.2
	N-4	34.0	43.0
	N-1'	69.3	66.0
	N-4'	59.8	55.3
Sungucine (**8**)	N-1	134.4	138.1
	N-4	52.5	49.1
	N-1'	135.4	137.6
	N-4'	42.9	46.4

Cartesian coordinates (Angstroms) of main conformers of *Strychnos* alkaloids **1-8**, optimized at the M06-2X/pecG-2 level in the liquid phase of particular solvent, modulated within the IEF-PCM scheme:

Compound: **1a**

symmetry c1

O	-2.537602000	2.124823000	0.435724000
O	1.999823000	3.293873000	0.192048000
N	1.194140000	1.191775000	0.523897000
N	-1.340750000	-2.607299000	0.318975000
C	-0.251390000	-2.068670000	-0.533485000
C	-0.796673000	-1.407362000	-1.790544000
C	-1.709965000	-0.250170000	-1.376666000
C	-0.797165000	0.823828000	-0.775309000
C	-0.006006000	0.330625000	0.437210000
C	2.339244000	0.446561000	0.177454000
C	3.640619000	0.888474000	-0.012837000
C	4.594728000	-0.054046000	-0.385970000
C	4.264506000	-1.391300000	-0.555849000
C	2.956169000	-1.819140000	-0.346329000
C	1.995807000	-0.895451000	0.018411000
C	0.552193000	-1.115960000	0.377911000
C	0.382969000	-1.817807000	1.735673000
C	-1.082202000	-2.226612000	1.714670000
C	-2.686543000	-2.245795000	-0.131339000
C	-2.785475000	-0.773437000	-0.436782000
C	-3.693874000	0.024615000	0.109586000
C	-3.689972000	1.510044000	-0.121719000
C	-1.404153000	2.183835000	-0.409364000
C	-0.376715000	3.030025000	0.372129000
C	1.056189000	2.541463000	0.340116000
H	0.413490000	-2.888033000	-0.815833000
H	-1.332058000	-2.139763000	-2.391926000
H	0.032332000	-1.042020000	-2.396730000
H	-2.194356000	0.175951000	-2.259649000
H	-0.060654000	1.044864000	-1.555390000
H	-0.616819000	0.461851000	1.332310000
H	3.895799000	1.925332000	0.119983000
H	5.613766000	0.268766000	-0.544709000

H	5.024381000	-2.102601000	-0.844488000
H	2.698416000	-2.863492000	-0.462141000
H	1.028110000	-2.696320000	1.761950000
H	0.641871000	-1.174578000	2.574725000
H	-1.296494000	-3.053883000	2.389811000
H	-1.714441000	-1.381534000	2.013555000
H	-3.396603000	-2.525114000	0.644756000
H	-2.936296000	-2.833414000	-1.015953000
H	-4.438526000	-0.385723000	0.781071000
H	-3.746695000	1.739796000	-1.191832000
H	-4.544624000	1.975894000	0.361932000
H	-1.686137000	2.696760000	-1.334142000
H	-0.375906000	4.066264000	0.052047000
H	-0.693810000	3.007759000	1.416555000

Compound: **2a**

symmetry c1

O	-3.593563000	2.012614000	0.103353000
O	0.866581000	3.468238000	0.175942000
N	0.159397000	1.348686000	0.604982000
N	-2.108815000	-2.621980000	0.489155000
C	-0.976730000	-2.076818000	-0.301471000
C	-1.436734000	-1.553594000	-1.654010000
C	-2.454698000	-0.433181000	-1.423419000
C	-1.670449000	0.744152000	-0.833785000
C	-0.971943000	0.401897000	0.483029000
C	1.373278000	0.653257000	0.424986000
C	2.654517000	1.181498000	0.321021000
C	3.709490000	0.295323000	0.122036000
C	3.472655000	-1.086654000	0.028935000
C	2.185757000	-1.581714000	0.149509000
C	1.128575000	-0.709485000	0.348641000
C	-0.324130000	-1.004522000	0.597143000
C	-0.581907000	-1.609064000	1.987156000
C	-2.010478000	-2.117298000	1.866312000
C	-3.424053000	-2.388681000	-0.110924000
C	-3.581949000	-0.954195000	-0.544954000
C	-4.585077000	-0.180268000	-0.150771000
C	-4.649071000	1.280029000	-0.501905000
C	-2.391280000	2.083791000	-0.639476000
C	-1.496241000	3.058223000	0.155823000
C	-0.040582000	2.665874000	0.298029000
H	-0.238585000	-2.868067000	-0.452401000
H	-1.865426000	-2.364896000	-2.239499000
H	-0.576572000	-1.179514000	-2.209371000
H	-2.876600000	-0.110935000	-2.379549000
H	-0.876527000	0.953039000	-1.558992000
H	-1.672667000	0.561300000	1.304515000
H	2.808840000	2.242427000	0.393707000
O	5.002182000	0.681144000	0.013950000
C	5.285182000	2.063470000	0.122928000
H	4.785594000	2.628903000	-0.664545000
H	6.359650000	2.160878000	0.015962000
H	4.977800000	2.449325000	1.095383000
O	4.520689000	-1.953120000	-0.130135000
C	5.038962000	-1.988473000	-1.454666000
H	5.394156000	-1.004458000	-1.758342000

H	5.867237000	-2.690271000	-1.449945000
H	4.271960000	-2.332640000	-2.150890000
H	2.041281000	-2.652738000	0.095849000
H	0.109955000	-2.436584000	2.146206000
H	-0.444865000	-0.886438000	2.789510000
H	-2.240072000	-2.902304000	2.585409000
H	-2.718293000	-1.296148000	2.033776000
H	-4.187547000	-2.655093000	0.617538000
H	-3.551324000	-3.058086000	-0.962947000
H	-5.364326000	-0.587858000	0.482020000
H	-4.618856000	1.423027000	-1.588029000
H	-5.571971000	1.722431000	-0.136061000
H	-2.615849000	2.501775000	-1.625755000
H	-1.525329000	4.062471000	-0.252976000
H	-1.911401000	3.101106000	1.164597000

Compound: **3a**

symmetry c1

O	2.132880000	2.604049000	-0.462163000
O	-2.517072000	3.154757000	-0.212222000
N	-1.421603000	1.186872000	-0.529453000
N	1.617475000	-2.316183000	-0.259389000
C	0.431352000	-1.820825000	0.589078000
C	0.900916000	-1.090650000	1.828816000
C	1.644046000	0.174850000	1.393122000
C	0.593305000	1.109688000	0.784481000
C	-0.114435000	0.506655000	-0.430221000
C	-2.451382000	0.288430000	-0.185125000
C	-3.804837000	0.540055000	-0.011663000
C	-4.616193000	-0.526754000	0.362309000
C	-4.099356000	-1.801466000	0.550269000
C	-2.741606000	-2.039191000	0.358362000
C	-1.923158000	-0.988665000	-0.007596000
C	-0.458191000	-1.005003000	-0.359615000
C	-0.191010000	-1.708314000	-1.702450000
C	1.308230000	-1.901505000	-1.681295000
C	2.908402000	-1.687735000	0.186460000
C	2.761193000	-0.217587000	0.441086000
C	3.554153000	0.678733000	-0.131373000
C	3.364472000	2.155608000	0.080602000
C	1.014231000	2.533270000	0.402803000
C	-0.126250000	3.233959000	-0.366319000
C	-1.475660000	2.544616000	-0.348289000
H	-0.069626000	-2.750319000	0.838110000
H	1.528206000	-1.745664000	2.429995000
H	0.032528000	-0.836514000	2.435854000
H	2.080741000	0.670947000	2.263547000
H	-0.168718000	1.234296000	1.560652000
H	0.475445000	0.720675000	-1.322675000
H	-4.205322000	1.527925000	-0.158521000
H	-5.672761000	-0.353057000	0.507748000
H	-4.752296000	-2.611661000	0.839196000
H	-2.337385000	-3.034548000	0.487906000
H	-0.698919000	-2.671795000	-1.709839000
H	-0.534300000	-1.122852000	-2.552446000
H	1.689902000	-2.696132000	-2.309832000
H	1.827726000	-0.968697000	-1.895211000
H	3.638561000	-1.906227000	-0.587259000

H	3.180311000	-2.236414000	1.084220000
H	4.337693000	0.354558000	-0.805238000
H	3.414073000	2.404856000	1.146350000
H	4.143049000	2.717304000	-0.427957000
H	1.244882000	3.086474000	1.317950000
H	-0.279259000	4.252162000	-0.025535000
H	0.189421000	3.281563000	-1.410274000
O	1.721143000	-3.668895000	-0.163801000

Compound: **4a**

symmetry c1

O	3.396548000	2.333304000	-0.146976000
O	-1.126580000	3.568057000	-0.227535000
N	-0.303733000	1.483275000	-0.613730000
N	2.155362000	-2.455134000	-0.387341000
C	0.979944000	-1.843411000	0.397339000
C	1.433073000	-1.280378000	1.726984000
C	2.390227000	-0.115724000	1.458938000
C	1.548146000	1.007629000	0.844899000
C	0.875343000	0.605026000	-0.468811000
C	-1.477721000	0.725253000	-0.424170000
C	-2.787477000	1.182085000	-0.340990000
C	-3.791700000	0.241666000	-0.130613000
C	-3.478733000	-1.124058000	-0.006257000
C	-2.165584000	-1.547826000	-0.104060000
C	-1.159866000	-0.618997000	-0.313784000
C	0.310346000	-0.839179000	-0.551498000
C	0.596836000	-1.482480000	-1.920007000
C	2.043306000	-1.903082000	-1.792037000
C	3.476753000	-2.061312000	0.211961000
C	3.521657000	-0.606754000	0.571987000
C	4.485470000	0.195629000	0.139343000
C	4.494978000	1.666306000	0.453925000
C	2.205397000	2.373577000	0.617221000
C	1.254357000	3.292424000	-0.179644000
C	-0.176450000	2.815895000	-0.327817000
H	0.327078000	-2.700338000	0.526385000
H	1.899137000	-2.063198000	2.321938000
H	0.557957000	-0.939008000	2.279002000
H	2.811198000	0.246971000	2.400085000
H	0.743313000	1.192936000	1.563623000
H	1.568619000	0.789152000	-1.290826000
H	-3.000997000	2.230643000	-0.438781000
O	-5.103266000	0.555107000	-0.040674000
C	-5.465334000	1.916214000	-0.184215000
H	-5.172431000	2.296038000	-1.163376000
H	-5.006315000	2.527243000	0.593694000
H	-6.544419000	1.952780000	-0.087420000
O	-4.478137000	-2.043172000	0.157440000
C	-5.010952000	-2.085921000	1.476566000
H	-5.420243000	-1.117237000	1.760659000

H	-5.801562000	-2.829642000	1.471819000
H	-4.236098000	-2.380315000	2.186554000
H	-1.960511000	-2.607649000	-0.026457000
H	-0.044073000	-2.354105000	-2.047349000
H	0.423417000	-0.795127000	-2.744971000
H	2.710360000	-1.047422000	-1.886120000
H	2.363466000	-2.701724000	-2.449243000
H	4.236690000	-2.335095000	-0.514133000
H	3.581130000	-2.704237000	1.081796000
H	5.271699000	-0.197597000	-0.493323000
H	4.483334000	1.832040000	1.536762000
H	5.390162000	2.136007000	0.056017000
H	2.430095000	2.819313000	1.590714000
H	1.219576000	4.295145000	0.232246000
H	1.668724000	3.366098000	-1.186863000
O	2.052334000	-3.811314000	-0.391814000

Compound: **5a**

symmetry c1

H	4.212244000	1.357220000	-0.463662000
H	2.494686000	-3.308394000	-0.403925000
O	0.637247000	4.366201000	0.950892000
H	4.871143000	-2.851794000	-0.977081000
C	4.675983000	-0.729700000	-0.754874000
C	3.845558000	0.345809000	-0.456368000
C	2.521932000	0.067539000	-0.146321000
C	2.040602000	-1.236784000	-0.127915000
C	2.875533000	-2.296797000	-0.422518000
C	4.204621000	-2.035430000	-0.741017000
N	1.479678000	0.957323000	0.180134000
C	0.176097000	0.241339000	0.202708000
C	0.583760000	-1.258463000	0.301210000
C	0.315541000	3.076132000	0.612704000
O	-0.404967000	3.070547000	-0.598736000
C	-1.334165000	2.013788000	-0.749883000
C	-0.683502000	0.657161000	-1.000331000
C	-4.453581000	1.081271000	-1.835323000
C	-4.110772000	0.081079000	-0.775346000
C	-2.964318000	-0.567200000	-0.563866000
C	-1.735764000	-0.382379000	-1.446126000
C	-0.167534000	-2.222742000	-0.614219000
C	-0.997506000	-1.688297000	-1.750338000
C	0.458639000	-1.756214000	1.759524000
O	-0.042395000	-3.411834000	-0.439235000
C	-0.996270000	-2.082530000	2.139390000
N	-1.930563000	-1.115248000	1.588885000
C	-2.884705000	-1.549778000	0.586145000
C	-2.455945000	-0.151090000	2.526719000
H	5.709912000	-0.537431000	-1.002828000
H	-0.343121000	0.510301000	1.113810000
H	-1.952365000	2.287969000	-1.601619000
H	-0.005257000	0.772802000	-1.851153000
H	-2.099772000	-0.013734000	-2.403479000
H	-1.665273000	-2.482021000	-2.078779000
H	-0.293914000	-1.496442000	-2.566644000
H	1.073224000	-2.644983000	1.892043000
H	0.872185000	-0.977999000	2.404160000
H	-1.238592000	-3.075053000	1.763846000
H	-1.085707000	-2.129327000	3.227042000

H	-3.887401000	-1.682093000	1.011555000
H	-2.575677000	-2.527653000	0.214706000
H	-4.921148000	-0.134170000	-0.085439000
H	-3.726105000	1.129149000	-2.640678000
H	-5.424407000	0.846651000	-2.269994000
H	-2.982563000	0.638762000	1.990085000
H	-1.642802000	0.310408000	3.088971000
H	-3.155493000	-0.599469000	3.245380000
H	-4.536165000	2.080472000	-1.403263000
H	-1.980525000	1.957487000	0.133541000
C	1.618838000	2.280121000	0.396169000
H	1.568233000	4.492544000	0.713444000
O	2.681849000	2.877690000	0.377172000
H	-0.301259000	2.640423000	1.405338000

Compound: **5b**

symmetry c1

H	4.424831000	-0.306266000	-0.740416000
H	1.192810000	-3.825104000	0.630500000
O	2.345516000	4.039963000	0.047057000
H	3.559360000	-4.414522000	0.148197000
C	4.123584000	-2.401850000	-0.323122000
C	3.731384000	-1.072560000	-0.441395000
C	2.408712000	-0.764778000	-0.154964000
C	1.501917000	-1.747981000	0.229571000
C	1.904597000	-3.064636000	0.341850000
C	3.228761000	-3.389726000	0.065259000
N	1.772810000	0.491176000	-0.158661000
C	0.326841000	0.350188000	0.145734000
C	0.124087000	-1.160597000	0.474039000
C	1.518290000	2.944661000	0.022653000
O	0.554899000	3.120827000	-0.988929000
C	-0.679714000	2.455489000	-0.778227000
C	-0.530080000	0.955021000	-0.982921000
C	-4.530742000	-0.838466000	-2.203877000
C	-4.212611000	-0.280549000	-0.850986000
C	-3.056272000	0.225598000	-0.416453000
C	-1.827549000	0.203501000	-1.333380000
C	-0.850862000	-1.924225000	-0.434654000
C	-1.393291000	-1.238593000	-1.653294000
C	-0.354278000	-1.351600000	1.927840000
O	-1.154460000	-3.057488000	-0.153298000
C	-1.790064000	-0.859176000	2.132653000
N	-1.932391000	0.551228000	1.789406000
C	-3.058842000	0.887425000	0.945715000
C	-1.804293000	1.423411000	2.937703000
H	5.148777000	-2.664779000	-0.540767000
H	0.132158000	0.901760000	1.054403000
H	-1.372113000	2.874007000	-1.506325000
H	0.066063000	0.859714000	-1.894183000
H	-2.153583000	0.644864000	-2.277593000
H	-2.198281000	-1.851020000	-2.045295000
H	-0.593487000	-1.208854000	-2.399490000
H	-0.297781000	-2.409743000	2.177615000
H	0.334919000	-0.819425000	2.586165000
H	-2.460518000	-1.446668000	1.505196000
H	-2.096934000	-1.042589000	3.167947000

H	-3.035205000	1.971798000	0.805630000
H	-4.021248000	0.665758000	1.429382000
H	-5.046181000	-0.270038000	-0.157453000
H	-3.809211000	-0.551935000	-2.965052000
H	-4.574730000	-1.929019000	-2.177576000
H	-2.647101000	1.326879000	3.636331000
H	-0.888900000	1.187406000	3.480191000
H	-1.745295000	2.463088000	2.615049000
H	-5.512948000	-0.492937000	-2.522549000
H	-1.050655000	2.680045000	0.226678000
C	2.388948000	1.687470000	-0.242733000
H	3.194222000	3.756637000	-0.325549000
O	3.572514000	1.837029000	-0.499555000
H	1.009190000	2.853304000	0.989479000

Compound: **5c**

symmetry c1

H	4.454181000	0.247631000	-0.615466000
H	1.437978000	-3.705137000	-0.611600000
O	2.015660000	4.117231000	1.056570000
H	3.800392000	-3.926404000	-1.361446000
C	4.258433000	-1.860155000	-1.032106000
C	3.805383000	-0.610393000	-0.620133000
C	2.482472000	-0.510493000	-0.215447000
C	1.637577000	-1.615240000	-0.211618000
C	2.099977000	-2.851317000	-0.618685000
C	3.423030000	-2.968275000	-1.035536000
N	1.770606000	0.626394000	0.224535000
C	0.318604000	0.321037000	0.308572000
C	0.280003000	-1.233642000	0.344068000
C	1.309960000	2.991468000	0.718521000
O	0.577477000	3.226383000	-0.463313000
C	-0.629645000	2.498675000	-0.574974000
C	-0.436957000	1.008340000	-0.840224000
C	-4.593911000	1.434994000	-2.037445000
C	-4.138998000	1.037586000	-0.668367000
C	-2.946281000	0.598651000	-0.264654000
C	-1.786236000	0.367400000	-1.217979000
C	-0.820211000	-1.899146000	-0.485353000
C	-1.551491000	-1.110460000	-1.538533000
C	0.136515000	-1.742000000	1.789941000
O	-0.972336000	-3.099038000	-0.402503000
C	-1.179992000	-1.232589000	2.410465000
N	-2.206104000	-1.058653000	1.389121000
C	-2.742660000	0.281907000	1.199015000
C	-3.224376000	-2.088878000	1.371287000
H	5.283967000	-1.962265000	-1.356657000
H	-0.040684000	0.701961000	1.259886000
H	-1.172340000	2.944640000	-1.406594000
H	0.197544000	0.912082000	-1.726429000
H	-2.059658000	0.841281000	-2.157502000
H	-2.482633000	-1.632129000	-1.753072000
H	-0.931138000	-1.176709000	-2.438834000
H	0.158008000	-2.829117000	1.754845000
H	0.990072000	-1.414124000	2.384374000
H	-1.516526000	-1.927972000	3.181140000
H	-1.008440000	-0.282603000	2.915310000

H	-2.043334000	0.996793000	1.633349000
H	-3.689567000	0.417079000	1.735193000
H	-4.905755000	1.123723000	0.095862000
H	-5.441356000	0.819603000	-2.340928000
H	-3.827258000	1.344285000	-2.800185000
H	-3.851858000	-1.963222000	0.488754000
H	-2.752860000	-3.067907000	1.311170000
H	-3.863544000	-2.050125000	2.261641000
H	-4.945419000	2.467201000	-2.030664000
H	-1.229827000	2.646491000	0.330150000
C	2.304030000	1.847105000	0.434377000
H	2.929215000	3.970931000	0.768226000
O	3.492724000	2.105086000	0.354400000
H	0.619750000	2.745081000	1.531501000

Compound: **5d**

symmetry c1

H	4.454286000	0.247844000	-0.615150000
H	1.438193000	-3.705005000	-0.611819000
O	2.015395000	4.117420000	1.056380000
H	3.800668000	-3.926151000	-1.361489000
C	4.258626000	-1.859915000	-1.031952000
C	3.805504000	-0.610184000	-0.619930000
C	2.482552000	-0.510349000	-0.215390000
C	1.637703000	-1.615143000	-0.211669000
C	2.100170000	-2.851166000	-0.618787000
C	3.423265000	-2.968051000	-1.035543000
N	1.770582000	0.626486000	0.224613000
C	0.318607000	0.321040000	0.308579000
C	0.280078000	-1.233642000	0.343969000
C	1.309714000	2.991572000	0.718484000
O	0.577077000	3.226533000	-0.463192000
C	-0.629895000	2.498597000	-0.574867000
C	-0.437019000	1.008302000	-0.840177000
C	-4.593730000	1.435238000	-2.037314000
C	-4.139026000	1.037551000	-0.668224000
C	-2.946338000	0.598483000	-0.264572000
C	-1.786289000	0.367321000	-1.217924000
C	-0.820007000	-1.899191000	-0.485550000
C	-1.551614000	-1.110524000	-1.538509000
C	0.136589000	-1.742080000	1.789831000
O	-0.971860000	-3.099131000	-0.402868000
C	-1.180004000	-1.232960000	2.410408000
N	-2.206085000	-1.059003000	1.389010000
C	-2.742678000	0.281566000	1.199038000
C	-3.224417000	-2.089172000	1.371136000
H	5.284196000	-1.961960000	-1.356410000
H	-0.040665000	0.701893000	1.259929000
H	-1.172663000	2.944525000	-1.406454000
H	0.197467000	0.912080000	-1.726397000
H	-2.059710000	0.841245000	-2.157413000
H	-2.482821000	-1.632220000	-1.752729000
H	-0.931571000	-1.176853000	-2.439021000
H	0.158268000	-2.829192000	1.754743000
H	0.990096000	-1.414062000	2.384260000
H	-1.516482000	-1.928602000	3.180857000
H	-1.008658000	-0.283064000	2.915492000

H	-2.043398000	0.996448000	1.633447000
H	-3.689589000	0.416622000	1.735253000
H	-4.905746000	1.123705000	0.096030000
H	-5.441928000	0.820819000	-2.340622000
H	-4.943932000	2.467890000	-2.030725000
H	-3.864128000	-2.049877000	2.261076000
H	-3.851364000	-1.963989000	0.488148000
H	-2.752927000	-3.068255000	1.311844000
H	-3.827240000	1.343462000	-2.800092000
H	-1.230101000	2.646227000	0.330276000
C	2.303884000	1.847253000	0.434404000
H	2.929165000	3.970657000	0.768921000
O	3.492568000	2.105317000	0.354493000
H	0.619617000	2.745051000	1.531545000

Compound: **5e**

symmetry c1

H	4.477168000	0.109161000	-0.348671000
H	1.458759000	-3.812145000	0.067343000
O	1.956993000	4.177112000	0.383648000
H	3.903169000	-4.137591000	-0.202373000
C	4.326999000	-2.039474000	-0.299161000
C	3.827195000	-0.742810000	-0.262488000
C	2.456300000	-0.585469000	-0.105332000
C	1.607814000	-1.679194000	0.002597000
C	2.119580000	-2.963708000	-0.022678000
C	3.490333000	-3.139860000	-0.177126000
N	1.710475000	0.608910000	-0.021532000
C	0.254003000	0.320934000	0.049586000
C	0.170548000	-1.234931000	0.181260000
C	1.258121000	3.022092000	0.144617000
O	0.616178000	3.098441000	-1.107185000
C	-0.623703000	2.425497000	-1.168369000
C	-0.503277000	0.902057000	-1.151730000
C	-4.812032000	-0.299732000	-1.823408000
C	-4.217914000	0.153914000	-0.527567000
C	-2.936556000	0.404133000	-0.253224000
C	-1.880978000	0.250553000	-1.354429000
C	-0.729270000	-1.969889000	-0.826385000
C	-1.639483000	-1.207100000	-1.751576000
C	-0.353432000	-1.660742000	1.571824000
O	-0.723084000	-3.175561000	-0.839898000
C	-1.860334000	-1.461474000	1.816075000
N	-2.367670000	-0.147288000	2.154570000
C	-2.617469000	0.883580000	1.148299000
C	-1.809283000	0.425523000	3.357248000
H	5.390554000	-2.185590000	-0.421539000
H	-0.110286000	0.751027000	0.977480000
H	-1.076968000	2.721830000	-2.112135000
H	0.089026000	0.635839000	-2.033215000
H	-2.301201000	0.739126000	-2.234894000
H	-2.561345000	-1.779206000	-1.835051000
H	-1.158013000	-1.236147000	-2.733308000
H	-0.146077000	-2.725968000	1.670107000
H	0.234010000	-1.148245000	2.336106000
H	-2.431028000	-1.836108000	0.965109000
H	-2.121582000	-2.109713000	2.654535000

H	-1.792631000	1.600950000	1.125744000
H	-3.475465000	1.459716000	1.501836000
H	-4.928138000	0.284362000	0.282377000
H	-5.710673000	0.274173000	-2.045020000
H	-4.131130000	-0.205847000	-2.665235000
H	-0.804679000	0.853457000	3.212969000
H	-2.454949000	1.228501000	3.713596000
H	-1.743977000	-0.333207000	4.135681000
H	-5.116421000	-1.346194000	-1.757465000
H	-1.271449000	2.785999000	-0.365523000
C	2.246941000	1.842605000	0.089234000
H	2.888967000	3.981721000	0.203142000
O	3.442664000	2.078885000	0.091138000
H	0.508099000	2.888682000	0.931920000

Compound: **5f**

symmetry c1

H	4.633460000	-0.030702000	-0.280456000
H	1.437180000	-3.815933000	0.039893000
O	2.356200000	4.042281000	0.970538000
H	3.865929000	-4.247723000	-0.166751000
C	4.386019000	-2.170388000	-0.242760000
C	3.944438000	-0.852775000	-0.210082000
C	2.579541000	-0.634569000	-0.076682000
C	1.678770000	-1.688715000	0.004051000
C	2.134155000	-2.994109000	-0.020556000
C	3.498433000	-3.232434000	-0.143253000
N	1.890386000	0.591885000	0.033266000
C	0.429677000	0.359957000	0.172894000
C	0.252024000	-1.188637000	0.087033000
C	1.567501000	2.969429000	0.641613000
O	0.744545000	3.310120000	-0.447058000
C	-0.498493000	2.640148000	-0.479309000
C	-0.367741000	1.171199000	-0.858697000
C	-4.671397000	0.877890000	-1.931346000
C	-4.109837000	0.805564000	-0.545564000
C	-2.839239000	0.636238000	-0.172522000
C	-1.737487000	0.578175000	-1.226913000
C	-0.535610000	-1.704862000	-1.131953000
C	-1.505119000	-0.799973000	-1.850605000
C	-0.476940000	-1.801626000	1.321264000
O	-0.411203000	-2.852225000	-1.477276000
C	-2.010281000	-1.885742000	1.331463000
N	-2.725302000	-0.781301000	1.951170000
C	-2.551969000	0.528671000	1.321422000
C	-4.126195000	-1.143063000	2.097468000
H	5.444228000	-2.363857000	-0.344142000
H	0.172627000	0.665971000	1.182293000
H	-1.096399000	3.154475000	-1.228926000
H	0.219693000	1.140650000	-1.781485000
H	-2.087386000	1.211632000	-2.040231000
H	-2.433399000	-1.355546000	-1.981831000
H	-1.079038000	-0.668273000	-2.848946000
H	-0.120737000	-2.828424000	1.376763000
H	-0.129034000	-1.296862000	2.223250000
H	-2.398845000	-2.091747000	0.322161000
H	-2.265240000	-2.767696000	1.917835000

H	-1.552861000	0.876453000	1.554046000
H	-3.225455000	1.207898000	1.842783000
H	-4.848811000	0.899269000	0.244155000
H	-5.478747000	0.153397000	-2.039800000
H	-5.107636000	1.860439000	-2.116659000
H	-4.202909000	-2.025655000	2.730289000
H	-4.668797000	-0.328941000	2.574480000
H	-4.613290000	-1.369634000	1.139240000
H	-3.940641000	0.675428000	-2.708806000
H	-1.004599000	2.750425000	0.486564000
C	2.484045000	1.785176000	0.259206000
H	3.249649000	3.840243000	0.654031000
O	3.684417000	1.979729000	0.176505000
H	0.938029000	2.708056000	1.500500000

Compound: **6a**

symmetry c1

O	0.497681000	-1.552634000	-1.855179000
N	0.736809000	-0.085625000	-0.066150000
H	-0.589819000	3.916100000	0.214356000
C	-0.471922000	3.201419000	1.032517000
C	-1.851048000	2.699808000	1.519487000
N	-2.431426000	1.612366000	0.725861000
C	-1.578992000	0.422307000	0.828267000
C	-0.176249000	0.795462000	0.473704000
C	1.902929000	0.608976000	-0.324787000
C	3.106514000	0.172403000	-0.870502000
C	4.121352000	1.104299000	-1.005227000
C	3.941376000	2.438399000	-0.613827000
C	2.741231000	2.869592000	-0.079109000
C	1.698077000	1.952182000	0.073389000
C	0.353737000	2.040262000	0.575609000
H	-1.766936000	2.376653000	-1.148899000
C	-2.684087000	2.024920000	-0.655203000
C	-3.258819000	0.881592000	-1.477959000
C	-2.295353000	-0.300759000	-1.454029000
C	-2.053679000	-0.771915000	-0.014917000
C	-0.950727000	-1.841653000	0.084005000
C	0.433149000	-1.437818000	-0.457925000
H	0.009672000	3.745491000	1.845773000
H	-2.559090000	3.527431000	1.533172000
H	-1.752980000	2.333657000	2.542212000
H	-1.606816000	0.107885000	1.877622000
H	3.248313000	-0.850521000	-1.191121000
H	5.068196000	0.797134000	-1.425307000
H	4.755434000	3.138404000	-0.736223000
H	2.609383000	3.901185000	0.218203000
H	-3.409332000	1.221723000	-2.501574000
H	-2.659189000	-1.129059000	-2.061371000
H	-1.350164000	0.014191000	-1.898443000
C	-3.334741000	-1.339137000	0.628015000
H	-0.841387000	-2.111168000	1.136012000
H	-1.236678000	-2.740782000	-0.460266000
H	-4.234989000	0.595287000	-1.084212000
H	-3.376752000	2.866106000	-0.620205000
C	-4.093416000	-2.360494000	-0.210942000
H	-3.450241000	-3.174234000	-0.545154000

H	-4.901736000	-2.798903000	0.371116000
H	-4.535975000	-1.902173000	-1.094097000
H	-3.994993000	-0.507096000	0.869804000
H	-3.054051000	-1.789115000	1.582686000
H	1.051108000	-2.318471000	-2.058610000
C	1.484210000	-2.402037000	0.131610000
O	1.694805000	-2.210346000	1.414806000
O	2.022004000	-3.244769000	-0.540585000
C	2.649368000	-3.086798000	2.033543000
H	2.318503000	-4.117066000	1.939125000
H	2.690967000	-2.788731000	3.073027000
H	3.618494000	-2.965194000	1.557762000

Compound: **6b**

symmetry c1

O	0.990284000	1.046642000	2.109144000
N	0.805817000	0.045397000	0.035720000
H	0.297380000	-4.101743000	0.162398000
C	-0.027143000	-3.457885000	-0.657557000
C	-1.559580000	-3.276832000	-0.602372000
N	-2.001993000	-2.110996000	0.172577000
C	-1.501616000	-0.907306000	-0.509577000
C	-0.019216000	-0.998967000	-0.358949000
C	2.103329000	-0.453937000	0.078150000
C	3.296693000	0.185993000	0.392554000
C	4.458942000	-0.564340000	0.346560000
C	4.434237000	-1.923393000	0.004388000
C	3.244757000	-2.558839000	-0.300974000
C	2.056925000	-1.823872000	-0.265745000
C	0.676827000	-2.145468000	-0.515490000
H	-3.744170000	-3.021193000	0.818667000
C	-3.449885000	-2.101191000	0.314936000
C	-3.873982000	-0.882481000	1.113034000
C	-3.517072000	0.383771000	0.345923000
C	-2.036114000	0.470849000	-0.060563000
C	-1.190948000	0.958415000	1.119217000
C	0.321337000	1.122858000	0.865472000
H	0.232614000	-3.979897000	-1.578862000
H	-2.011123000	-4.162875000	-0.160291000
H	-1.949912000	-3.192903000	-1.627941000
H	-1.753405000	-0.998396000	-1.579522000
H	3.318526000	1.230499000	0.668040000
H	5.402492000	-0.092764000	0.580725000
H	5.360870000	-2.478813000	-0.021219000
H	3.231272000	-3.607338000	-0.566177000
H	-3.380628000	-0.906743000	2.085856000
H	-4.119990000	0.403672000	-0.565989000
H	-3.788140000	1.270990000	0.918352000
C	-1.907749000	1.409630000	-1.278307000
H	-1.566781000	1.903730000	1.510172000
H	-1.253255000	0.227481000	1.926469000
H	-4.947904000	-0.910620000	1.291590000
H	-3.947757000	-2.090396000	-0.669873000
C	-2.307041000	2.859919000	-1.028303000
H	-3.347491000	2.935946000	-0.715616000

H	-1.699813000	3.339523000	-0.261000000
H	-2.194630000	3.440459000	-1.942287000
H	-2.544260000	1.003545000	-2.065931000
H	-0.889038000	1.367737000	-1.664335000
H	0.881593000	1.899545000	2.547165000
C	0.667431000	2.520084000	0.306862000
O	1.057713000	2.540243000	-0.949181000
O	0.589489000	3.492793000	1.014776000
C	1.360581000	3.836092000	-1.487706000
H	1.647122000	3.666387000	-2.517524000
H	2.175191000	4.286252000	-0.927679000
H	0.480296000	4.471078000	-1.430493000

Compound: **6c**

symmetry c1

O	0.967795000	-2.211507000	-1.700601000
N	1.006325000	-0.351416000	-0.335859000
H	0.991607000	3.863127000	-0.159016000
C	0.848666000	3.171354000	0.673871000
C	-0.631052000	3.141542000	1.072722000
N	-1.428604000	2.327688000	0.156722000
C	-1.081584000	0.912317000	0.311294000
C	0.383190000	0.808767000	0.063178000
C	2.372968000	-0.130689000	-0.341900000
C	3.443509000	-0.978535000	-0.625777000
C	4.719396000	-0.451863000	-0.540199000
C	4.939233000	0.887606000	-0.186141000
C	3.880114000	1.726543000	0.098313000
C	2.576457000	1.224451000	0.028773000
C	1.286080000	1.796320000	0.281177000
H	-3.066205000	3.579736000	0.367316000
C	-2.860843000	2.511635000	0.343924000
C	-3.633844000	1.840113000	-0.807204000
C	-2.755519000	0.826070000	-1.540527000
C	-1.957981000	-0.043283000	-0.561361000
C	-1.071558000	-1.001524000	-1.374603000
C	0.239398000	-1.501054000	-0.738621000
H	1.437170000	3.555580000	1.507772000
H	-1.035143000	4.151970000	1.066089000
H	-0.724966000	2.756778000	2.100713000
H	-1.249054000	0.620880000	1.364105000
H	3.281714000	-2.007451000	-0.902504000
H	5.565838000	-1.089878000	-0.750868000
H	5.951441000	1.262286000	-0.132633000
H	4.051243000	2.757613000	0.377167000
H	-3.969516000	2.593081000	-1.517722000
H	-3.356824000	0.197116000	-2.196889000
H	-2.045986000	1.356970000	-2.174042000
C	-2.913980000	-0.825036000	0.367376000
H	-0.735029000	-0.503322000	-2.284622000
H	-1.635760000	-1.880572000	-1.682966000
H	-4.528731000	1.357013000	-0.415596000
H	-3.176492000	2.106417000	1.317996000
C	-3.984968000	-1.660773000	-0.324007000
H	-3.561913000	-2.395253000	-1.007648000

H	-4.678573000	-1.039059000	-0.887489000
H	-4.563356000	-2.206427000	0.419020000
H	-3.402389000	-0.112691000	1.033538000
H	-2.323705000	-1.466632000	1.023194000
H	0.681585000	-3.132383000	-1.658378000
C	-0.038661000	-2.461842000	0.432059000
O	0.025508000	-1.890996000	1.617470000
O	-0.311485000	-3.618212000	0.235003000
C	-0.302912000	-2.729748000	2.735478000
H	-0.209888000	-2.101409000	3.611729000
H	0.387746000	-3.566437000	2.782753000
H	-1.320049000	-3.099469000	2.631011000

Compound: **7a**

symmetry c1

C	0.949017000	-1.873204000	1.768991000
C	1.445054000	-0.770807000	1.049296000
C	0.616158000	-0.194957000	0.095132000
C	-0.645833000	-0.699397000	-0.144895000
C	-1.119049000	-1.780482000	0.579853000
C	-0.325289000	-2.390048000	1.545364000
C	-1.710064000	-0.189337000	-1.076859000
C	-2.761905000	-1.333979000	-0.979922000
N	-2.445829000	-2.075486000	0.252344000
C	-2.242343000	1.194642000	-0.622440000
C	-3.366640000	1.216940000	0.422603000
C	-4.476226000	0.137246000	0.154317000
C	-4.200288000	-0.786093000	-1.043017000
C	-1.246947000	0.076830000	-2.536384000
C	-1.537373000	1.554595000	-2.791008000
N	-2.638754000	1.850953000	-1.874910000
C	-2.880713000	3.273737000	-1.699456000
C	-3.739150000	3.523681000	-0.499671000
C	-3.972997000	2.603427000	0.422517000
C	-2.852749000	-3.471960000	0.295028000
O	1.788300000	-2.387630000	2.701502000
H	0.955588000	0.669001000	-0.463370000
H	-0.686456000	-3.227969000	2.118096000
H	-2.636565000	-1.998276000	-1.841724000
H	-1.390449000	1.751240000	-0.208809000
C	-2.806026000	0.945028000	1.844221000
O	-5.744830000	0.738689000	-0.104723000
H	-4.544032000	-0.501052000	1.029223000
O	-4.406729000	-0.155819000	-2.282921000
C	-5.210469000	-1.939449000	-1.005488000
H	-1.814878000	-0.546270000	-3.222625000
H	-0.193073000	-0.156578000	-2.665664000
H	-1.820411000	1.773558000	-3.818069000
H	-0.665913000	2.171021000	-2.534514000
H	-3.357448000	3.667413000	-2.597270000
H	-1.927220000	3.812176000	-1.583435000
H	-4.170150000	4.511269000	-0.405166000
H	-4.613540000	2.842082000	1.264825000
H	-2.505417000	-3.923523000	1.218849000
H	-3.935029000	-3.558156000	0.289816000

H	-2.443889000	-4.034628000	-0.551064000
C	-1.639474000	1.820560000	2.288135000
C	-6.777757000	0.706503000	0.751686000
O	-7.824375000	1.166177000	0.375863000
C	-6.580559000	0.133319000	2.125387000
O	-5.094353000	-2.717366000	-2.070322000
O	-5.997559000	-2.139764000	-0.115818000
C	-5.981006000	-3.840728000	-2.126382000
C	1.315053000	-3.419027000	3.548838000
H	-3.636464000	1.091166000	2.536905000
H	-2.527171000	-0.104635000	1.922380000
H	-3.898010000	0.694932000	-2.276453000
H	-1.492730000	1.725280000	3.362236000
H	-0.711181000	1.519168000	1.805240000
H	-1.818403000	2.872457000	2.064384000
H	-6.347492000	-0.926620000	2.059034000
H	-5.764292000	0.633474000	2.641775000
H	-7.500270000	0.273218000	2.680219000
H	-5.810513000	-4.491874000	-1.272278000
H	-7.012885000	-3.500846000	-2.123332000
H	-5.748816000	-4.354658000	-3.050537000
H	0.435509000	-3.092099000	4.103568000
H	1.075682000	-4.315157000	2.976596000
H	2.122938000	-3.634700000	4.239008000
C	2.559643000	5.499342000	-0.403779000
C	3.598940000	5.181937000	-1.296508000
C	4.141804000	3.914193000	-1.337706000
C	3.639162000	2.933677000	-0.472410000
C	2.595513000	3.274687000	0.411397000
C	2.044822000	4.555425000	0.461261000
C	3.960110000	1.547888000	-0.251984000
C	3.097345000	1.104747000	0.724877000
N	2.292962000	2.149973000	1.125593000
C	5.049335000	0.831939000	-0.994120000
C	3.840163000	-1.869884000	-0.498605000
C	5.155372000	-2.531275000	-0.822625000
C	5.992351000	-2.081655000	-1.755058000
C	5.654861000	-0.864207000	-2.593709000
C	7.304675000	-2.750626000	-2.054169000
C	7.414242000	-3.229987000	-3.503145000
H	2.156263000	6.501761000	-0.396359000
H	3.974620000	5.947088000	-1.960472000
H	4.938961000	3.682724000	-2.031186000
H	1.246027000	4.794670000	1.148858000

H	1.619712000	2.077029000	1.872031000
H	5.698969000	0.289810000	-0.301157000
H	5.681071000	1.585846000	-1.464835000
H	5.437217000	-3.394960000	-0.227904000
H	5.390379000	-1.190002000	-3.603736000
H	6.545071000	-0.238847000	-2.697811000
H	8.115484000	-2.044684000	-1.855092000
H	7.439719000	-3.592721000	-1.375702000
H	8.357787000	-3.746609000	-3.667193000
H	6.602752000	-3.916693000	-3.742514000
H	7.366822000	-2.396346000	-4.202077000
N	4.564377000	-0.050073000	-2.066507000
C	3.476593000	-0.925901000	-1.649261000
H	2.604897000	-0.327313000	-1.416686000
H	3.216151000	-1.540225000	-2.513208000
C	2.857831000	-0.260167000	1.349287000
C	3.938615000	-1.276470000	0.926200000
H	3.061339000	-2.637042000	-0.464195000
C	2.927429000	0.005743000	2.868020000
H	4.909364000	-0.817708000	1.083556000
H	3.890809000	-2.111727000	1.619939000
O	2.041279000	0.545572000	3.483467000
O	4.079677000	-0.347023000	3.415287000
C	4.206847000	-0.091414000	4.818077000
H	5.192189000	-0.445075000	5.094847000
H	4.110847000	0.972880000	5.014607000
H	3.437488000	-0.632730000	5.362811000

Compound: **7b**

symmetry c1

C	-1.095952000	-1.611561000	-0.791630000
C	-1.216099000	-0.954880000	0.445264000
C	-0.041108000	-0.748584000	1.179410000
C	1.185229000	-1.156863000	0.719601000
C	1.277524000	-1.811666000	-0.503991000
C	0.147848000	-2.050159000	-1.268849000
C	2.543316000	-1.083246000	1.362934000
C	3.485405000	-1.686554000	0.254877000
N	2.577968000	-2.151699000	-0.792939000
C	2.948560000	0.321978000	1.817726000
C	3.276384000	1.350190000	0.719859000
C	3.789697000	0.647955000	-0.570475000
C	4.532914000	-0.663944000	-0.295633000
C	2.641815000	-1.857936000	2.693836000
C	3.728915000	-1.128494000	3.505050000
N	4.096014000	0.035325000	2.685987000
C	4.559567000	1.192343000	3.427005000
C	4.787247000	2.329939000	2.479542000
C	4.268408000	2.357889000	1.260756000
C	2.886332000	-3.273386000	-1.656933000
O	-2.220181000	-1.800668000	-1.522492000
H	-0.109292000	-0.274317000	2.153092000
H	0.230478000	-2.552766000	-2.218365000
H	4.067163000	-2.520979000	0.653259000
H	2.129669000	0.721842000	2.429808000
C	2.011894000	2.145300000	0.296664000
O	4.639936000	1.539273000	-1.287473000
H	2.929760000	0.386544000	-1.183131000
O	5.618370000	-0.521967000	0.572861000
C	5.078547000	-1.228579000	-1.608988000
H	2.860753000	-2.909904000	2.531766000
H	1.684159000	-1.795379000	3.207036000
H	4.610435000	-1.739869000	3.688623000
H	3.334220000	-0.806036000	4.472164000
H	5.487612000	0.935030000	3.938437000
H	3.835656000	1.479548000	4.204736000
H	5.414128000	3.141941000	2.822270000
H	4.481705000	3.195725000	0.609422000
H	3.872985000	-3.657212000	-1.407852000
H	2.162416000	-4.077165000	-1.509577000

H	2.885798000	-2.987485000	-2.708802000
C	1.271796000	2.890807000	1.400761000
C	4.385753000	1.963793000	-2.539919000
O	5.254584000	2.578102000	-3.100281000
C	3.042988000	1.694123000	-3.160338000
O	5.990890000	-2.167889000	-1.401912000
O	4.698032000	-0.905202000	-2.704712000
C	6.516095000	-2.791141000	-2.579312000
C	-2.130119000	-2.441130000	-2.780273000
H	2.338787000	2.868488000	-0.453526000
H	1.322127000	1.461662000	-0.202732000
H	5.234306000	-0.321383000	1.468748000
H	0.689432000	2.217369000	2.028850000
H	1.957619000	3.443094000	2.042042000
H	0.576352000	3.603279000	0.960640000
H	2.233907000	2.045023000	-2.524465000
H	3.009975000	2.213440000	-4.110552000
H	2.916884000	0.626487000	-3.321073000
H	6.999547000	-2.049393000	-3.208952000
H	7.232465000	-3.524629000	-2.231258000
H	5.714691000	-3.270779000	-3.136727000
H	-3.142542000	-2.473073000	-3.168556000
H	-1.494781000	-1.877599000	-3.464305000
H	-1.747383000	-3.456822000	-2.678797000
C	-7.173726000	-3.415264000	-1.256003000
C	-7.636575000	-2.160626000	-1.689169000
C	-6.938138000	-1.004935000	-1.403128000
C	-5.746034000	-1.095567000	-0.673029000
C	-5.304645000	-2.364412000	-0.244633000
C	-6.007089000	-3.535263000	-0.527656000
C	-4.786881000	-0.124893000	-0.215592000
C	-3.805387000	-0.836895000	0.431864000
N	-4.146256000	-2.170238000	0.453677000
C	-4.939229000	1.349098000	-0.444713000
C	-2.039363000	1.934708000	0.035004000
C	-2.385192000	3.378595000	0.289560000
C	-3.327747000	4.036286000	-0.382087000
C	-4.121021000	3.358840000	-1.482648000
C	-3.659949000	5.476540000	-0.111478000
C	-3.469587000	6.371569000	-1.337847000
H	-7.741922000	-4.301902000	-1.498261000
H	-8.554101000	-2.104687000	-2.257191000
H	-7.304376000	-0.046688000	-1.746079000
H	-5.649624000	-4.497884000	-0.190671000

H	-3.533353000	-2.897476000	0.785545000
H	-4.841335000	1.898587000	0.496796000
H	-5.959077000	1.528275000	-0.786398000
H	-1.855133000	3.883613000	1.092029000
H	-3.769241000	3.721053000	-2.452814000
H	-5.169628000	3.657467000	-1.406516000
H	-4.701256000	5.544129000	0.215000000
H	-3.042255000	5.839037000	0.709960000
H	-3.681383000	7.410440000	-1.093032000
H	-2.445016000	6.308758000	-1.703416000
H	-4.134800000	6.079688000	-2.149088000
N	-4.042929000	1.901688000	-1.472113000
C	-2.650321000	1.501403000	-1.301955000
H	-2.565961000	0.431263000	-1.452415000
H	-2.083510000	1.984376000	-2.100296000
C	-2.504248000	-0.426445000	1.097661000
C	-2.423809000	1.106814000	1.279927000
H	-0.954309000	1.849028000	-0.073770000
C	-2.621842000	-1.097042000	2.486517000
H	-3.365231000	1.449387000	1.695440000
H	-1.677384000	1.315511000	2.047789000
O	-2.222641000	-2.204594000	2.739899000
O	-3.282765000	-0.347551000	3.359491000
C	-3.528021000	-0.944852000	4.637569000
H	-4.071294000	-0.203687000	5.210304000
H	-4.120516000	-1.847950000	4.517211000
H	-2.586622000	-1.189690000	5.121669000

Compound: **7c**

symmetry c1

C	0.339428000	-2.783643000	-0.148394000
C	0.977724000	-1.537402000	-0.280286000
C	0.213363000	-0.459333000	-0.724966000
C	-1.126411000	-0.610272000	-1.010843000
C	-1.736925000	-1.848490000	-0.859144000
C	-1.015495000	-2.956680000	-0.431663000
C	-2.122176000	0.378247000	-1.557494000
C	-3.472247000	-0.428748000	-1.515458000
N	-3.073277000	-1.790890000	-1.173820000
C	-2.184334000	1.715918000	-0.816313000
C	-2.775157000	1.706007000	0.604913000
C	-3.745532000	0.506003000	0.782413000
C	-4.510593000	0.153708000	-0.499190000
C	-1.793845000	0.860700000	-2.985690000
C	-2.450356000	2.250163000	-3.096355000
N	-2.970379000	2.530152000	-1.750187000
C	-3.026909000	3.931958000	-1.383331000
C	-3.489859000	4.050983000	0.037194000
C	-3.439199000	3.036526000	0.887904000
C	-3.808699000	-2.963573000	-1.597731000
O	1.120155000	-3.806879000	0.276589000
H	0.687804000	0.505940000	-0.855868000
H	-1.496039000	-3.914422000	-0.313304000
H	-3.969720000	-0.409006000	-2.487839000
H	-1.162850000	2.114357000	-0.764346000
C	-1.663724000	1.522688000	1.672425000
O	-4.665074000	0.797534000	1.831472000
H	-3.157543000	-0.372455000	1.040563000
O	-5.208420000	1.233293000	-1.048549000
C	-5.532455000	-0.945455000	-0.205432000
H	-2.143053000	0.158668000	-3.738469000
H	-0.713448000	0.945401000	-3.086435000
H	-3.264320000	2.284160000	-3.818144000
H	-1.711020000	3.001763000	-3.385553000
H	-3.721261000	4.443188000	-2.050821000
H	-2.046047000	4.414769000	-1.511491000
H	-3.891219000	5.005114000	0.350759000
H	-3.805998000	3.166696000	1.898106000
H	-4.173856000	-3.543879000	-0.749709000
H	-4.664265000	-2.653058000	-2.193149000

H	-3.178806000	-3.604666000	-2.217249000
C	-0.576602000	2.589008000	1.712531000
C	-4.775921000	0.054715000	2.948942000
O	-5.693021000	0.302170000	3.686565000
C	-3.750690000	-1.004839000	3.244191000
O	-6.438947000	-1.048222000	-1.167290000
O	-5.499056000	-1.673139000	0.753448000
C	-7.401158000	-2.095613000	-1.002420000
C	0.533316000	-5.079354000	0.475854000
H	-2.164530000	1.502778000	2.643053000
H	-1.202500000	0.542747000	1.532145000
H	-4.520125000	1.849531000	-1.413187000
H	0.084328000	2.541922000	0.847735000
H	-0.998119000	3.591824000	1.764640000
H	0.047848000	2.440588000	2.591469000
H	-3.961209000	-1.399678000	4.230880000
H	-3.818902000	-1.801563000	2.507750000
H	-2.742097000	-0.599228000	3.220788000
H	-8.053394000	-2.036437000	-1.864672000
H	-6.899974000	-3.060216000	-0.965563000
H	-7.962354000	-1.945136000	-0.084365000
H	0.125931000	-5.471279000	-0.456022000
H	1.331519000	-5.726201000	0.822995000
H	-0.253647000	-5.032031000	1.228612000
C	3.304728000	4.288024000	1.803440000
C	3.734199000	4.541082000	0.488435000
C	3.762614000	3.537498000	-0.458520000
C	3.352427000	2.247118000	-0.094450000
C	2.919671000	2.021131000	1.226298000
C	2.894398000	3.028253000	2.189899000
C	3.294537000	0.989675000	-0.792646000
C	2.841791000	0.067279000	0.119478000
N	2.602359000	0.695728000	1.320799000
C	3.724699000	0.862825000	-2.233293000
C	4.771973000	-1.923362000	-1.404673000
C	5.591556000	-1.344969000	-0.274372000
C	6.202653000	-0.165506000	-0.336908000
C	6.072250000	0.729166000	-1.548205000
C	7.051381000	0.364278000	0.784312000
C	6.303243000	1.392101000	1.638259000
H	3.298758000	5.093737000	2.523508000
H	4.050284000	5.539032000	0.220052000
H	4.107070000	3.748072000	-1.462310000
H	2.569525000	2.827485000	3.201108000

H	2.263227000	0.212904000	2.137822000
H	3.707904000	1.874431000	-2.642602000
H	3.005628000	0.293158000	-2.820097000
H	5.729041000	-1.955082000	0.609240000
H	7.047074000	0.760549000	-2.048549000
H	5.860632000	1.752215000	-1.231865000
H	7.376164000	-0.462500000	1.415923000
H	7.951943000	0.825355000	0.370495000
H	5.966091000	2.240985000	1.043190000
H	5.419507000	0.935672000	2.084298000
H	6.939102000	1.769414000	2.437870000
N	5.058376000	0.309347000	-2.505658000
C	5.101153000	-1.134202000	-2.678339000
H	4.424180000	-1.413812000	-3.486361000
H	6.114043000	-1.387562000	-2.997605000
C	2.496555000	-1.401863000	-0.033857000
C	3.227468000	-2.060142000	-1.222371000
H	5.136295000	-2.941117000	-1.558884000
C	2.805994000	-2.119318000	1.294329000
H	2.971455000	-3.114852000	-1.179771000
H	2.744000000	-1.678564000	-2.118996000
O	2.278922000	-1.829940000	2.342844000
O	3.701234000	-3.086280000	1.188869000
C	3.967528000	-3.821739000	2.385113000
H	4.698318000	-4.573804000	2.113932000
H	4.362829000	-3.161437000	3.152644000
H	3.051729000	-4.286427000	2.742593000

Compound: **8a**

symmetry c1

N	2.525927000	-1.394799000	-0.153798000
C	3.312970000	-0.488126000	-1.013471000
C	5.346153000	0.190511000	0.442109000
N	6.232357000	1.128665000	-0.251444000
C	6.897630000	0.262004000	-1.226092000
C	5.785561000	-0.637444000	-1.804025000
C	4.784596000	-0.796567000	-0.641155000
C	4.661918000	-2.184235000	-0.068011000
C	5.660505000	-3.092668000	0.219751000
C	5.324212000	-4.302101000	0.822396000
C	4.000152000	-4.577046000	1.134754000
C	2.981508000	-3.670321000	0.854549000
C	3.334296000	-2.473372000	0.245215000
C	4.250463000	0.857876000	1.244037000
C	3.452111000	1.771096000	0.308716000
C	2.849169000	0.962021000	-0.890197000
C	1.354719000	1.005768000	-0.836037000
C	3.161079000	4.867689000	0.678585000
C	4.286262000	4.135881000	0.015063000
C	4.410102000	2.825668000	-0.178211000
C	5.576047000	2.255053000	-0.937432000
C	1.182676000	-1.277605000	0.051579000
C	0.575953000	0.002030000	-0.433691000
N	-1.349546000	0.250174000	1.067468000
C	-4.057091000	0.279445000	-0.892516000
C	-2.692974000	-0.339467000	1.212105000
N	-5.413321000	-0.264514000	-0.662419000
C	-0.916527000	0.188870000	-0.327659000
C	-1.779362000	-0.915290000	-0.936828000
C	-3.119824000	-0.759777000	-0.219755000
C	-3.995975000	-1.985433000	-0.190674000
C	-3.648132000	-3.292319000	0.086337000
C	-4.646411000	-4.261784000	0.133067000
C	-5.971135000	-3.908593000	-0.085967000
C	-6.338883000	-2.594126000	-0.358398000
C	-5.328736000	-1.643289000	-0.410203000
C	-3.686031000	0.645632000	1.822900000
C	-3.597592000	1.967790000	1.056528000
C	-3.926809000	1.742073000	-0.461426000
C	-5.163581000	2.492753000	-0.862212000

C	-2.823578000	4.884111000	0.488044000
C	-1.884790000	3.812330000	0.952480000
C	-2.204537000	2.552537000	1.230827000
C	-1.177467000	1.547396000	1.700868000
C	-6.548974000	0.455906000	-0.863162000
C	-6.337026000	1.906893000	-1.069201000
H	3.146077000	-0.804473000	-2.048060000
H	5.982863000	-0.398872000	1.104464000
H	7.414275000	0.850990000	-1.980575000
H	7.638135000	-0.345049000	-0.703921000
H	5.302619000	-0.154714000	-2.653885000
H	6.162516000	-1.596392000	-2.151662000
H	6.691914000	-2.866602000	-0.020081000
H	6.093910000	-5.025401000	1.048476000
H	3.746787000	-5.517211000	1.603510000
H	1.955589000	-3.886311000	1.094356000
H	4.681710000	1.454860000	2.046562000
H	3.615596000	0.097420000	1.698816000
H	2.639034000	2.225493000	0.867354000
H	3.147730000	1.460183000	-1.812340000
H	0.892126000	1.953314000	-1.095958000
H	2.327747000	4.222135000	0.941266000
H	2.784268000	5.652452000	0.022362000
H	3.506857000	5.359792000	1.588455000
H	5.089385000	4.766475000	-0.353455000
H	5.216774000	1.945305000	-1.929535000
H	6.324167000	3.027510000	-1.109384000
H	-3.881901000	0.240826000	-1.972059000
H	-2.632049000	-1.239687000	1.827612000
H	-1.139106000	1.134297000	-0.844915000
H	-1.867799000	-0.823863000	-2.017577000
H	-1.351361000	-1.884822000	-0.699615000
H	-2.614824000	-3.556685000	0.270845000
H	-4.390022000	-5.289814000	0.343028000
H	-6.739107000	-4.667900000	-0.046092000
H	-7.364969000	-2.318360000	-0.527646000
H	-3.459466000	0.814420000	2.874548000
H	-4.691176000	0.227137000	1.780385000
H	-4.331644000	2.663962000	1.460667000
H	-3.101675000	2.160021000	-1.038111000
H	-5.078779000	3.569101000	-0.946194000
H	-7.226077000	2.464256000	-1.324275000
H	-2.760029000	5.022043000	-0.593739000
H	-2.573642000	5.840505000	0.943889000

H	-3.858770000	4.655971000	0.733162000
H	-0.844715000	4.103679000	1.064315000
H	-0.168057000	1.908163000	1.501655000
H	-1.251296000	1.413472000	2.781933000
O	0.536430000	-2.140413000	0.621878000
O	-7.668608000	-0.030203000	-0.832972000

Compound: **8b**

symmetry c1

N	-2.626664000	-1.253340000	-0.539735000
C	-3.410595000	-0.663682000	0.565190000
C	-5.223406000	0.768439000	-0.607910000
N	-6.089607000	1.455356000	0.353239000
C	-6.921776000	0.361025000	0.856468000
C	-5.955146000	-0.819177000	1.085972000
C	-4.859790000	-0.624446000	0.017175000
C	-4.802912000	-1.678615000	-1.058069000
C	-5.842428000	-2.272937000	-1.745048000
C	-5.552985000	-3.177286000	-2.763641000
C	-4.233192000	-3.464749000	-3.083547000
C	-3.173227000	-2.872505000	-2.402497000
C	-3.481794000	-1.980105000	-1.384733000
C	-3.999210000	1.561835000	-1.010446000
C	-3.221389000	1.932797000	0.256286000
C	-2.808541000	0.652412000	1.059515000
C	-1.315002000	0.540157000	1.112822000
C	-2.648534000	4.878762000	1.164406000
C	-3.884662000	4.074309000	1.420484000
C	-4.131179000	2.815072000	1.069790000
C	-5.408066000	2.130639000	1.471290000
C	-1.269369000	-1.215827000	-0.601990000
C	-0.604381000	-0.308807000	0.375487000
N	1.497123000	0.342087000	1.506659000
C	3.899104000	-0.605865000	-0.611624000
C	2.782035000	0.959610000	1.110547000
N	5.206929000	0.009688000	-0.929013000
C	0.903803000	-0.355676000	0.370307000
C	1.525612000	0.348859000	-0.836869000
C	2.960839000	0.612551000	-0.389648000
C	3.695085000	1.703231000	-1.126310000
C	3.253705000	2.962698000	-1.479432000
C	4.137518000	3.841728000	-2.099731000
C	5.446859000	3.452400000	-2.346772000
C	5.909812000	2.189021000	-1.991386000
C	5.011216000	1.323280000	-1.382396000
C	3.952337000	0.449175000	1.945506000
C	3.905817000	-1.079508000	1.966738000
C	3.994525000	-1.648501000	0.506192000
C	5.239993000	-2.466083000	0.323510000

C	3.398410000	-3.886669000	3.095959000
C	2.417435000	-2.754375000	3.115200000
C	2.625262000	-1.525189000	2.654802000
C	1.562928000	-0.450600000	2.727989000
C	6.376297000	-0.685425000	-0.941332000
C	6.300511000	-2.041696000	-0.353147000
H	-3.375519000	-1.377138000	1.394657000
H	-5.839770000	0.568148000	-1.486312000
H	-7.458237000	0.662148000	1.753493000
H	-7.656303000	0.102140000	0.092855000
H	-5.520576000	-0.772592000	2.084601000
H	-6.446465000	-1.784983000	0.994560000
H	-6.869351000	-2.038112000	-1.494755000
H	-6.355920000	-3.654326000	-3.306246000
H	-4.017168000	-4.166790000	-3.876184000
H	-2.150303000	-3.098518000	-2.647400000
H	-4.295446000	2.472245000	-1.529811000
H	-3.390849000	0.970599000	-1.695286000
H	-2.321317000	2.471638000	-0.024651000
H	-3.153304000	0.778397000	2.085510000
H	-0.785486000	1.232979000	1.756748000
H	-2.261504000	5.282323000	2.100298000
H	-2.873584000	5.732998000	0.524718000
H	-1.855530000	4.305020000	0.693918000
H	-4.665631000	4.595148000	1.965758000
H	-5.173224000	1.412296000	2.270064000
H	-6.100500000	2.852721000	1.901424000
H	3.566613000	-1.125721000	-1.515005000
H	2.708599000	2.045161000	1.204715000
H	1.191011000	-1.415613000	0.348019000
H	1.453928000	-0.236520000	-1.748858000
H	1.011534000	1.299083000	-0.990757000
H	2.235054000	3.265097000	-1.274868000
H	3.804165000	4.827846000	-2.387943000
H	6.126481000	4.141195000	-2.827963000
H	6.924098000	1.885574000	-2.182601000
H	3.894991000	0.833467000	2.962839000
H	4.887863000	0.814555000	1.522753000
H	4.761878000	-1.454607000	2.526208000
H	3.151913000	-2.325424000	0.368780000
H	5.263723000	-3.435286000	0.806050000
H	7.201139000	-2.629879000	-0.447941000
H	3.193859000	-4.568209000	2.267204000
H	3.329642000	-4.472422000	4.010952000

H	4.424649000	-3.539578000	2.995655000
H	1.443267000	-2.974989000	3.539763000
H	0.584320000	-0.891350000	2.915679000
H	1.768682000	0.223847000	3.561853000
O	-0.625587000	-1.846763000	-1.427687000
O	7.427461000	-0.224584000	-1.357460000

Compound: **8c**

symmetry c1

N	-2.783791000	-1.533874000	-0.450249000
C	-3.258498000	-0.968343000	0.829751000
C	-4.483520000	1.209839000	0.153790000
N	-4.999133000	1.936585000	1.317041000
C	-6.144349000	1.114490000	1.711945000
C	-5.655652000	-0.345162000	1.614647000
C	-4.618510000	-0.321037000	0.473194000
C	-4.994633000	-1.087164000	-0.768686000
C	-6.210798000	-1.138513000	-1.419544000
C	-6.315103000	-1.860328000	-2.605873000
C	-5.203194000	-2.509677000	-3.124017000
C	-3.968745000	-2.466146000	-2.481618000
C	-3.885742000	-1.748011000	-1.296390000
C	-3.074050000	1.599013000	-0.238825000
C	-2.163199000	1.403949000	0.977764000
C	-2.208823000	-0.078012000	1.487365000
C	-0.851075000	-0.693241000	1.337795000
C	-0.524792000	3.734102000	2.262462000
C	-1.950280000	3.369439000	2.541234000
C	-2.653572000	2.363869000	2.027745000
C	-4.070770000	2.097049000	2.451038000
C	-1.506400000	-1.950085000	-0.666271000
C	-0.501948000	-1.510995000	0.347550000
N	1.831659000	-1.613693000	1.187880000
C	3.861174000	-0.344424000	-1.259297000
C	2.262512000	-0.274914000	0.764530000
N	4.189850000	1.077443000	-1.502530000
C	0.899529000	-2.039833000	0.136814000
C	1.551900000	-1.540679000	-1.185433000
C	2.384772000	-0.307955000	-0.792869000
C	1.918464000	1.002267000	-1.373593000
C	0.630689000	1.477182000	-1.519948000
C	0.434664000	2.772995000	-1.991514000
C	1.527740000	3.569220000	-2.306744000
C	2.832626000	3.100064000	-2.178360000
C	3.006667000	1.803537000	-1.713399000
C	3.523635000	0.210021000	1.447161000
C	4.637626000	-0.810483000	1.203261000
C	4.872886000	-1.022828000	-0.331408000
C	6.260703000	-0.590260000	-0.702222000

C	6.133330000	-2.341947000	3.494700000
C	4.819765000	-2.698103000	2.871867000
C	4.188865000	-2.080089000	1.877137000
C	2.902538000	-2.621526000	1.320066000
C	5.462332000	1.526522000	-1.676859000
C	6.520937000	0.548062000	-1.334965000
H	-3.445523000	-1.815115000	1.497975000
H	-5.169341000	1.422280000	-0.668479000
H	-6.495508000	1.388921000	2.704098000
H	-6.956811000	1.285906000	1.004619000
H	-5.182763000	-0.655349000	2.546712000
H	-6.464240000	-1.044836000	1.416683000
H	-7.071562000	-0.623029000	-1.012326000
H	-7.261556000	-1.914414000	-3.123569000
H	-5.292475000	-3.067646000	-4.045142000
H	-3.109013000	-2.974247000	-2.881395000
H	-3.043317000	2.644304000	-0.544552000
H	-2.753878000	0.994190000	-1.088300000
H	-1.141134000	1.645016000	0.699659000
H	-2.440032000	-0.060666000	2.551731000
H	-0.101889000	-0.392846000	2.058976000
H	0.040173000	3.787719000	3.193350000
H	-0.467185000	4.722215000	1.804196000
H	-0.021096000	3.031826000	1.603420000
H	-2.458176000	4.013519000	3.252450000
H	-4.075795000	1.198155000	3.084213000
H	-4.436343000	2.913097000	3.072604000
H	3.906065000	-0.852962000	-2.227331000
H	1.452787000	0.415539000	0.995584000
H	0.836605000	-3.127828000	0.128909000
H	2.197669000	-2.323394000	-1.583909000
H	0.819297000	-1.317128000	-1.953088000
H	-0.214163000	0.843955000	-1.276672000
H	-0.568057000	3.156423000	-2.115262000
H	1.368026000	4.573737000	-2.671671000
H	3.679604000	3.711708000	-2.436034000
H	3.355799000	0.309928000	2.518899000
H	3.786666000	1.194195000	1.059696000
H	5.558096000	-0.448047000	1.650879000
H	4.807747000	-2.091146000	-0.537256000
H	7.073948000	-1.232001000	-0.385153000
H	7.526917000	0.868587000	-1.561370000
H	6.807078000	-3.198580000	3.467130000
H	6.000007000	-2.082768000	4.545676000

H	6.628632000	-1.509985000	3.003021000
H	4.337000000	-3.575549000	3.290773000
H	3.121326000	-3.069963000	0.340320000
H	2.531254000	-3.427241000	1.951093000
O	-1.188769000	-2.623395000	-1.635278000
O	5.729628000	2.661787000	-2.037228000

Compound: **8d**

symmetry c1

N	-2.783791000	-1.533874000	-0.450249000
C	-3.258498000	-0.968343000	0.829751000
C	-4.483520000	1.209839000	0.153790000
N	-4.999133000	1.936585000	1.317041000
C	-6.144349000	1.114490000	1.711945000
C	-5.655652000	-0.345162000	1.614647000
C	-4.618510000	-0.321037000	0.473194000
C	-4.994633000	-1.087164000	-0.768686000
C	-6.210798000	-1.138513000	-1.419544000
C	-6.315103000	-1.860328000	-2.605873000
C	-5.203194000	-2.509677000	-3.124017000
C	-3.968745000	-2.466146000	-2.481618000
C	-3.885742000	-1.748011000	-1.296390000
C	-3.074050000	1.599013000	-0.238825000
C	-2.163199000	1.403949000	0.977764000
C	-2.208823000	-0.078012000	1.487365000
C	-0.851075000	-0.693241000	1.337795000
C	-0.524792000	3.734102000	2.262462000
C	-1.950280000	3.369439000	2.541234000
C	-2.653572000	2.363869000	2.027745000
C	-4.070770000	2.097049000	2.451038000
C	-1.506400000	-1.950085000	-0.666271000
C	-0.501948000	-1.510995000	0.347550000
N	1.831659000	-1.613693000	1.187880000
C	3.861174000	-0.344424000	-1.259297000
C	2.262512000	-0.274914000	0.764530000
N	4.189850000	1.077443000	-1.502530000
C	0.899529000	-2.039833000	0.136814000
C	1.551900000	-1.540679000	-1.185433000
C	2.384772000	-0.307955000	-0.792869000
C	1.918464000	1.002267000	-1.373593000
C	0.630689000	1.477182000	-1.519948000
C	0.434664000	2.772995000	-1.991514000
C	1.527740000	3.569220000	-2.306744000
C	2.832626000	3.100064000	-2.178360000
C	3.006667000	1.803537000	-1.713399000
C	3.523635000	0.210021000	1.447161000
C	4.637626000	-0.810483000	1.203261000
C	4.872886000	-1.022828000	-0.331408000
C	6.260703000	-0.590260000	-0.702222000

C	6.133330000	-2.341947000	3.494700000
C	4.819765000	-2.698103000	2.871867000
C	4.188865000	-2.080089000	1.877137000
C	2.902538000	-2.621526000	1.320066000
C	5.462332000	1.526522000	-1.676859000
C	6.520937000	0.548062000	-1.334965000
H	-3.445523000	-1.815115000	1.497975000
H	-5.169341000	1.422280000	-0.668479000
H	-6.495508000	1.388921000	2.704098000
H	-6.956811000	1.285906000	1.004619000
H	-5.182763000	-0.655349000	2.546712000
H	-6.464240000	-1.044836000	1.416683000
H	-7.071562000	-0.623029000	-1.012326000
H	-7.261556000	-1.914414000	-3.123569000
H	-5.292475000	-3.067646000	-4.045142000
H	-3.109013000	-2.974247000	-2.881395000
H	-3.043317000	2.644304000	-0.544552000
H	-2.753878000	0.994190000	-1.088300000
H	-1.141134000	1.645016000	0.699659000
H	-2.440032000	-0.060666000	2.551731000
H	-0.101889000	-0.392846000	2.058976000
H	0.040173000	3.787719000	3.193350000
H	-0.467185000	4.722215000	1.804196000
H	-0.021096000	3.031826000	1.603420000
H	-2.458176000	4.013519000	3.252450000
H	-4.075795000	1.198155000	3.084213000
H	-4.436343000	2.913097000	3.072604000
H	3.906065000	-0.852962000	-2.227331000
H	1.452787000	0.415539000	0.995584000
H	0.836605000	-3.127828000	0.128909000
H	2.197669000	-2.323394000	-1.583909000
H	0.819297000	-1.317128000	-1.953088000
H	-0.214163000	0.843955000	-1.276672000
H	-0.568057000	3.156423000	-2.115262000
H	1.368026000	4.573737000	-2.671671000
H	3.679604000	3.711708000	-2.436034000
H	3.355799000	0.309928000	2.518899000
H	3.786666000	1.194195000	1.059696000
H	5.558096000	-0.448047000	1.650879000
H	4.807747000	-2.091146000	-0.537256000
H	7.073948000	-1.232001000	-0.385153000
H	7.526917000	0.868587000	-1.561370000
H	6.807078000	-3.198580000	3.467130000
H	6.000007000	-2.082768000	4.545676000

H	6.628632000	-1.509985000	3.003021000
H	4.337000000	-3.575549000	3.290773000
H	3.121326000	-3.069963000	0.340320000
H	2.531254000	-3.427241000	1.951093000
O	-1.188769000	-2.623395000	-1.635278000
O	5.729628000	2.661787000	-2.037228000

Compound: **8e**

symmetry c1

N	-2.626772000	-1.253114000	-0.539872000
C	-3.410601000	-0.663556000	0.565230000
C	-5.223429000	0.768499000	-0.607814000
N	-6.089555000	1.455526000	0.353301000
C	-6.921676000	0.361273000	0.856765000
C	-5.955079000	-0.818988000	1.086247000
C	-4.859813000	-0.624377000	0.017398000
C	-4.803078000	-1.678586000	-1.057835000
C	-5.842610000	-2.273106000	-1.744574000
C	-5.553227000	-3.177463000	-2.763192000
C	-4.233467000	-3.464732000	-3.083359000
C	-3.173461000	-2.872289000	-2.402519000
C	-3.481965000	-1.979927000	-1.384727000
C	-3.999259000	1.561856000	-1.010449000
C	-3.221286000	1.932809000	0.256175000
C	-2.808483000	0.652524000	1.059497000
C	-1.314942000	0.540075000	1.112877000
C	-2.648194000	4.878826000	1.164030000
C	-3.884350000	4.074454000	1.420299000
C	-4.130971000	2.815228000	1.069675000
C	-5.407859000	2.130910000	1.471291000
C	-1.269500000	-1.215578000	-0.602222000
C	-0.604444000	-0.308860000	0.375374000
N	1.497091000	0.341519000	1.506835000
C	3.899282000	-0.605605000	-0.611668000
C	2.781981000	0.959224000	1.110849000
N	5.207155000	0.010274000	-0.928409000
C	0.903755000	-0.355887000	0.370288000
C	1.525680000	0.348798000	-0.836760000
C	2.960836000	0.612551000	-0.389419000
C	3.694961000	1.703537000	-1.125794000
C	3.253413000	2.962913000	-1.478935000
C	4.137175000	3.842177000	-2.099000000
C	5.446640000	3.453169000	-2.345791000
C	5.909786000	2.189844000	-1.990378000
C	5.011250000	1.323878000	-1.381669000
C	3.952230000	0.448587000	1.945743000
C	3.905905000	-1.080085000	1.966382000
C	3.994591000	-1.648652000	0.505678000
C	5.239928000	-2.466317000	0.322465000

C	3.398724000	-3.887679000	3.094429000
C	2.417623000	-2.755487000	3.114196000
C	2.625339000	-1.526119000	2.654238000
C	1.563007000	-0.451629000	2.727907000
C	6.376388000	-0.685051000	-0.941426000
C	6.300462000	-2.041598000	-0.353976000
H	-3.375326000	-1.377072000	1.394645000
H	-5.839803000	0.568139000	-1.486189000
H	-7.458043000	0.662479000	1.753818000
H	-7.656312000	0.102318000	0.093275000
H	-5.520470000	-0.772439000	2.084868000
H	-6.446488000	-1.784769000	0.994977000
H	-6.869543000	-2.038464000	-1.494145000
H	-6.356239000	-3.654634000	-3.305571000
H	-4.017457000	-4.166749000	-3.876018000
H	-2.150561000	-3.098161000	-2.647651000
H	-4.295595000	2.472303000	-1.529712000
H	-3.391010000	0.970697000	-1.695456000
H	-2.321217000	2.471580000	-0.024906000
H	-3.153251000	0.778552000	2.085488000
H	-0.785297000	1.232701000	1.756893000
H	-2.873218000	5.732767000	0.523935000
H	-1.855159000	4.304869000	0.693861000
H	-2.261262000	5.282790000	2.099776000
H	-4.665234000	4.595388000	1.965606000
H	-5.173096000	1.412616000	2.270118000
H	-6.100266000	2.853070000	1.901325000
H	3.567145000	-1.125063000	-1.515416000
H	2.708463000	2.044739000	1.205309000
H	1.190822000	-1.415865000	0.347787000
H	1.454052000	-0.236596000	-1.748756000
H	1.011538000	1.298966000	-0.990794000
H	2.234681000	3.265172000	-1.274559000
H	3.803611000	4.828230000	-2.387197000
H	6.126230000	4.142114000	-2.826806000
H	6.924191000	1.886681000	-2.181403000
H	3.894662000	0.832464000	2.963227000
H	4.887754000	0.814348000	1.523325000
H	4.761961000	-1.455344000	2.525749000
H	3.151868000	-2.325406000	0.368065000
H	5.263672000	-3.435843000	0.804337000
H	7.200964000	-2.629917000	-0.449171000
H	4.424918000	-3.540401000	2.994371000
H	3.194282000	-4.568763000	2.265277000

H	3.329971000	-4.473924000	4.009101000
H	1.443530000	-2.976344000	3.538794000
H	0.584415000	-0.892508000	2.915387000
H	1.768644000	0.222568000	3.561998000
O	-0.625784000	-1.846406000	-1.428088000
O	7.427594000	-0.224104000	-1.357374000

Compound: **8f**

symmetry c1

N	2.586255000	1.277562000	-0.459212000
C	3.411931000	0.573984000	0.543998000
C	4.904388000	-0.826415000	-1.081759000
N	6.052050000	-1.603344000	-0.587757000
C	6.900389000	-0.646211000	0.116019000
C	5.951120000	0.324439000	0.823244000
C	4.778500000	0.453271000	-0.154980000
C	4.756479000	1.693741000	-1.015925000
C	5.792667000	2.366222000	-1.630571000
C	5.500054000	3.444869000	-2.463220000
C	4.182581000	3.824143000	-2.676813000
C	3.124971000	3.157250000	-2.062861000
C	3.436071000	2.094961000	-1.227594000
C	3.630582000	-1.661814000	-1.094550000
C	3.260517000	-2.005027000	0.349703000
C	2.788756000	-0.714529000	1.081947000
C	1.293673000	-0.623629000	1.088968000
C	3.220949000	-3.162355000	3.189384000
C	4.414078000	-3.132101000	2.283051000
C	4.450875000	-2.656923000	1.041530000
C	5.681259000	-2.796107000	0.178848000
C	1.231030000	1.248332000	-0.505283000
C	0.577405000	0.252580000	0.390614000
N	-1.525547000	-0.511268000	1.450941000
C	-3.916631000	0.583733000	-0.607378000
C	-2.803358000	-1.104153000	0.997231000
N	-5.218806000	-0.012623000	-0.979486000
C	-0.931129000	0.278457000	0.377083000
C	-1.535771000	-0.336231000	-0.886406000
C	-2.972659000	-0.643166000	-0.473201000
C	-3.695441000	-1.677438000	-1.297326000
C	-3.244373000	-2.903237000	-1.744203000
C	-4.119211000	-3.736339000	-2.436344000
C	-5.429385000	-3.336383000	-2.660864000
C	-5.902121000	-2.106928000	-2.211482000
C	-5.012453000	-1.286222000	-1.531461000
C	-3.983938000	-0.666834000	1.859004000
C	-3.947979000	0.856012000	1.999362000
C	-4.025312000	1.536341000	0.586550000
C	-5.272612000	2.361378000	0.456189000

C	-3.470766000	3.564313000	3.368449000
C	-2.482623000	2.440500000	3.297949000
C	-2.677215000	1.253189000	2.733591000
C	-1.607349000	0.183374000	2.729748000
C	-6.392765000	0.673717000	-0.943024000
C	-6.327295000	1.983179000	-0.256039000
H	3.530613000	1.268038000	1.382227000
H	5.133026000	-0.478198000	-2.089407000
H	7.583409000	-1.157453000	0.790056000
H	7.501667000	-0.107166000	-0.619807000
H	5.604138000	-0.118356000	1.758540000
H	6.403432000	1.287875000	1.050573000
H	6.818408000	2.061250000	-1.470198000
H	6.301193000	3.985224000	-2.945950000
H	3.966584000	4.659377000	-3.327558000
H	2.102958000	3.454840000	-2.221164000
H	3.791784000	-2.565535000	-1.680675000
H	2.823797000	-1.110002000	-1.575900000
H	2.426869000	-2.709972000	0.353480000
H	3.109386000	-0.798600000	2.119536000
H	0.759810000	-1.352741000	1.688737000
H	3.123435000	-4.143544000	3.652601000
H	2.294369000	-2.949450000	2.660319000
H	3.318847000	-2.439300000	4.001680000
H	5.324433000	-3.564265000	2.685831000
H	6.535193000	-3.101329000	0.780166000
H	5.501972000	-3.605594000	-0.534066000
H	-3.581319000	1.173307000	-1.465799000
H	-2.723250000	-2.193206000	1.007730000
H	-1.230099000	1.333567000	0.434367000
H	-1.461697000	0.318828000	-1.749648000
H	-1.012557000	-1.267850000	-1.108319000
H	-2.225296000	-3.215076000	-1.557008000
H	-3.778227000	-4.695356000	-2.797785000
H	-6.101891000	-3.989544000	-3.198408000
H	-6.916921000	-1.794696000	-2.385149000
H	-3.932457000	-1.128380000	2.844058000
H	-4.913388000	-1.004121000	1.401001000
H	-4.812124000	1.181166000	2.577205000
H	-3.184237000	2.224454000	0.509549000
H	-5.303555000	3.292685000	1.007926000
H	-7.230202000	2.572730000	-0.313179000
H	-3.250460000	4.326919000	2.618253000
H	-3.427496000	4.055125000	4.339322000

H	-4.492034000	3.225193000	3.209029000
H	-1.513562000	2.630634000	3.748271000
H	-0.633844000	0.615053000	2.960135000
H	-1.814560000	-0.555800000	3.506452000
O	0.577325000	1.958501000	-1.255906000
O	-7.439967000	0.237914000	-1.394782000
